# Orientational Behavior and Vibrational Response of Glycine at Aqueous Interfaces

**DOI:** 10.1021/acs.jpclett.3c02930

**Published:** 2024-02-15

**Authors:** Balázs Antalicz, Sanghamitra Sengupta, Aswathi Vilangottunjalil, Jan Versluis, Huib J. Bakker

**Affiliations:** Ultrafast Spectroscopy, AMOLF, Science Park 104, 1098 XG Amsterdam, The Netherlands

## Abstract

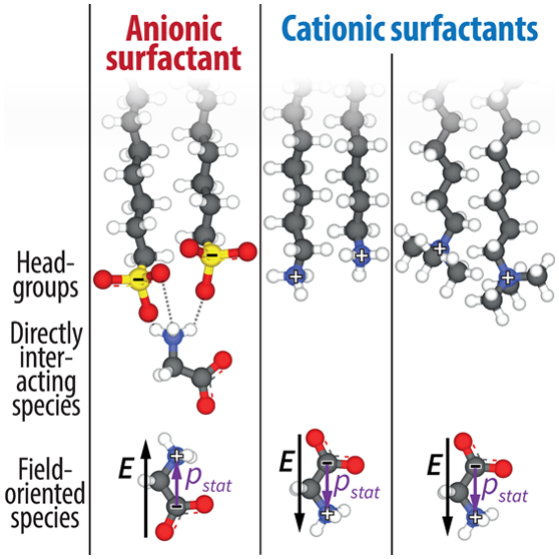

Aqueous glycine plays many different roles in living systems, from being a building block for proteins to being a neurotransmitter. To better understand its fundamental behavior, we study glycine’s orientational behavior near model aqueous interfaces, in the absence and presence of electric fields and biorelevant ions. To this purpose, we use a surface-specific technique called heterodyne-detected vibrational sum-frequency generation spectroscopy (HD-VSFG). Using HD-VSFG, we directly probe the symmetric and antisymmetric stretching vibrations of the carboxylate group of zwitterionic glycine. From their relative amplitudes, we infer the zwitterion’s orientation near surfactant-covered interfaces and find that it is governed by both electrostatic and surfactant-specific interactions. By introducing additional ions, we observe that the net orientation is altered by the enhanced ionic strength, indicating a change in the balance of the electrostatic and surfactant-specific interactions.

Glycine, the simplest amino acid,^1^ is essential in a multitude of biological systems. It is a building block for proteins and a crucial component in several metabolical pathways.^2,3^ Glycine additionally plays a regulatory role in immune function and in the determination of intracellular Ca^2+^ levels.^4^ It also serves as a major inhibitory neurotransmitter in the spinal cord and the brain stem.^5,6^ Considering the ubiquitous nature of glycine in biological systems, its physicochemical properties, which govern its biochemical behavior, have been thoroughly studied. Earlier studies investigated its structural^7^ and vibrational properties,^8−12^ and the properties of its aqueous solvation shell^13−18^ as well as some of its physicochemical properties at water/solid interfaces^19,20^ and surfactant-covered water/air interfaces.^21−23^ In addition, the specific interaction between metal ions and carboxylates/glycine has been the subject of numerous studies,^24−35^ many of which suggest that ion-specific interactions play a significant role. To better understand glycine’s behavior as a neurotransmitter, further studies are required, related to its behavior at aqueous interfaces and in the presence of ions.

In this work, we study glycine’s molecular-level behavior at water/air interfaces, with surface charges and additional ions present. To this purpose, we use heterodyne-detected vibrational sum-frequency generation spectroscopy (HD-VSFG). HD-VSFG is a uniquely surface-sensitive spectroscopic technique^36^ and has been utilized to study the effect of charged-surface induced electric fields on the orientation of dipolar molecules, like water^37^ and urea.^38^ Similarly to these studies, we create charged monolayers of anionic DS^–^ (dodecyl-sulfate, Na^+^ counterion) and cationic D[T]A^+^ (dodecyl-[trimethyl]ammonium, Br^–^ counterion); see the method description in the Supporting Information. By investigating the differences between glycine’s HD-VSFG signals with DS^–^/D(T)A^+^ monolayers present, we obtain information on the effect of electric fields on the orientation of glycine. The differences in glycine SFG signals with DA^+^/DTA^+^ monolayers provide information about how surfactant-specific hydrogen-bonding interactions impact the orientation of glycine. Finally, we also investigate the effect of the addition of different ions to the solution.

In Figure 1(a), we present the chemical structures of cationic, zwitterionic, and anionic glycine species. In Figure 1(b), we show the steady-state infrared absorption spectra (*A*) of these molecular structures, recorded in heavy water (D_2_O). The corresponding spectra in H_2_O are presented in the Supporting Information, see Figure S6(a). The characteristic vibrational modes in this spectral window include the C=O stretching vibration (ν^C=O^) of the cationic glycine species and the symmetric/antisymmetric stretching vibrations of the COO^–^ group  of the zwitterionic and anionic glycine species. We list the frequency assignment of the main spectral features in Table 1. We additionally decompose all displayed absorption spectra; see Figure S7 and Tables S2 and S3.

**Figure 1:**
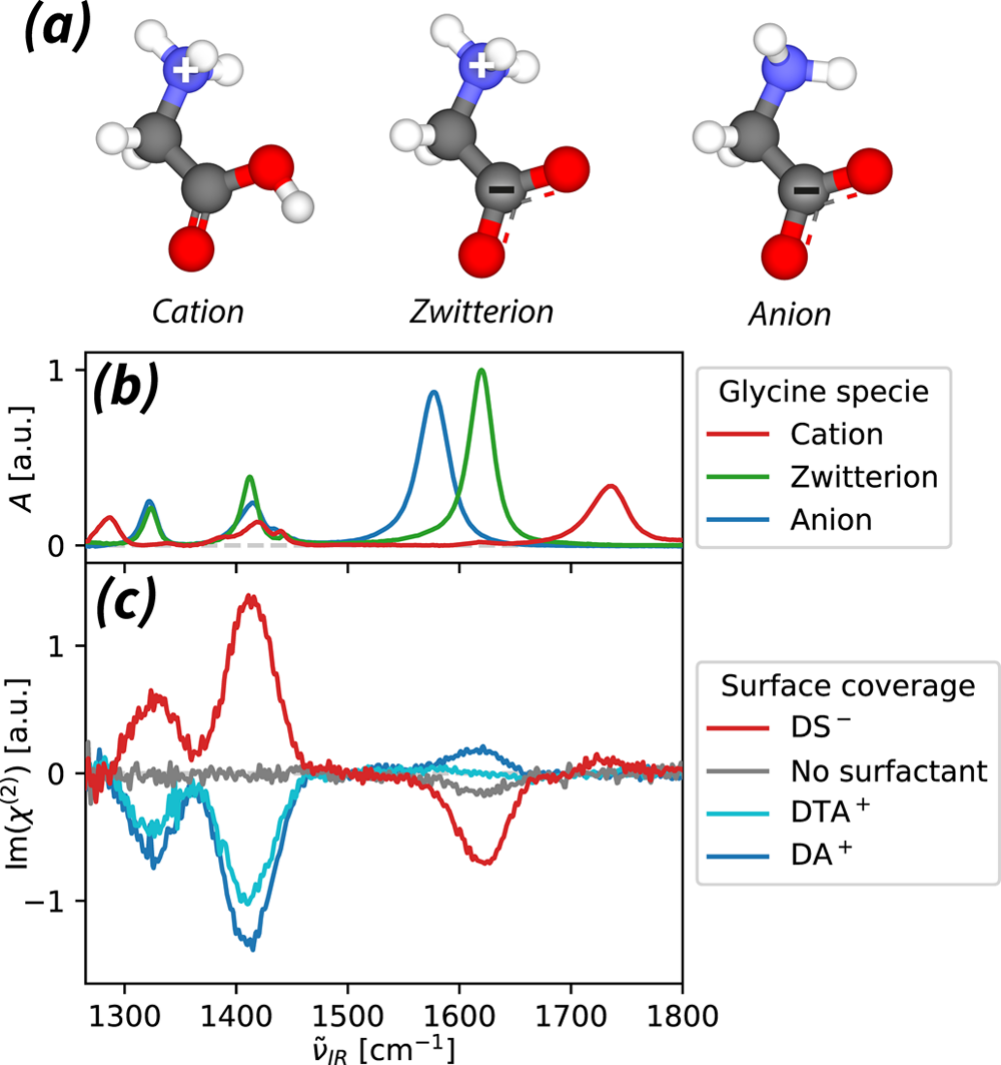
Comparison of chemical structures and vibrational features of different glycine species. (a) Left to right: chemical structures of cationic, zwitterionic, and anionic glycine. (b) Steady-state infrared absorption spectra (*A*) of glycine, recorded at acidic/neutral/basic conditions in heavy water (D_2_O), plotted in function of the spatial frequency of the exciting infrared light . In the presented spectra, we subtracted the infrared absorption of the solvent and normalized the signals to the sample thickness, see Supporting Information. The main vibrational features are assigned in Table 1. (c) HD-VSFG spectra (Im(χ^(2)^), SSP polarization^36^) of 1 M glycine solutions at neutral pD, at the neat D_2_O/air interface and in the presence of monolayers of charged surfactants. The above spectra are presented after subtracting the corresponding HD-VSFG spectra of neat and surfactant-covered D_2_O/air interfaces, see Figure S5.

**Table 1:** Assignment of the Main Vibrational^c^ Features of Different Glycine Species in D_2_O and H_2_O, Based on Earlier IR and Raman Studies^8−12^

ν̃_IR_^D_2_O^ [cm^–1^]	ν̃_IR_^H_2_O^ [cm^–1^]	Mode	Specie
1734	1740	ν^C=O^	cation
1577	1564	ν_as_^COO–^	anion
1620	1600^a^	ν_as_^COO–^	zwitterion
1412	1413	ν_s_^COO–^	zwitterion
1324^b^	1332^b^	ω^CH^_^2^_	zwitterion
1287	1261	ν^C–O^	cation

a From peak fitting: overlaps/mixes  (1633 cm^–1^).

b Possibly overlapping/mixing with other modes.

c Denoted modes: ν = stretching, δ = bending, ω = wagging. Vibration types: *s* = symmetric, *as* = anti/asymmetric.

In Figure 1(c), we show HD-VSFG spectra (Im(χ^(2)^), SSP polarization^36^) of a solution of 1 M glycine with and without added charged surfactants. For all samples, the observed glycine SFG signals appear at frequencies matching those of the zwitterionic form (, see Figure 1(b)).

At the neat D_2_O/air interface, we observe no signals corresponding to the zwitterion’s  and the  vibrations, and only a weak negative signal from the  vibration. At the DS^–^/D(T)A^+^-covered surfaces, we observe clearly positive/negative responses from the  and the  vibrations. In the case of DS^–^ coverage, we additionally observe a small signal at 1735 cm^–1^, which matches the ν^C=O^ vibration of cationic glycine. We make similar observations with H_2_O as a solvent, see Figure S6b. The main difference with the spectra measured in D_2_O is that the HD-VSFG response of the  vibration is much broader, probably due to mixing/coupling with the overlapping  vibration. Overall, in both solvents, we find that both the amplitude and the sign of the observable HD-VSFG spectral features of glycine are highly influenced by the charge of the surfactant monolayer.

To explain the origin of the observed SFG signals, we need to consider the balance of glycine species in bulk solutions, the role of electrostatics, and the connection between the molecular orientation and the observable SFG signals.

In a neutral glycine solution, the different glycine species equilibrate and form a buffer solution. This is because the acid dissociation constants^8^ of the cation (p*K*_a_^cation^ = 2.35) and the zwitterion (p*K*_a_^zwitterion^ = 10.00) are close enough to each other to allow a small portion of the zwitterions to react with water and to become anionic/cationic. In a 1 M glycine solution, the solution pH is thus pH = 1/2·(p*K*_a_^cation^ + p*K*_a_^zwitterion^) ≈ 6.2, and the ionic glycine species are present in a low concentration: 1 M ≈ 0.15 mM. The main fraction of glycine thus remains zwitterionic and is present mainly in the form of monomers.^39^ We confirm this by observing the HD-VSFG signals of glycine at different concentrations (Figures S8, S9, and S10). We find that these signals scale with glycine concentration and that the band frequencies do not change, indicating that all observed HD-VSFG signals originate from glycine monomers.

Next, we consider electrostatics. The densely packed DS^–^/D(T)A^+^ surfactant monolayers have a high surface-charge density ,^40,41^ which induces a strong electric field that penetrates the bulk of the solution.^42^ Because of ionic screening (*I* = *c*^surfactant^ + *c*^ion^ = 2.15 mM), this electric field decays with a Debye length of λ_D_ ≈ 4 nm (based on linearized Poisson–Boltzmann model, see the Supporting Information of an earlier work^38^). The electric field then orients the zwitterionic glycine species by interacting with its strong static dipole moment ( D).^15^

We connect the observed HD-VSFG signals with the zwitterion’s molecular orientation, using the results of previous theoretical works.^43−45^ These works describe the amplitude  of the Im (χ_ssp_^2^) contributions of *C*_2*v*_-symmetric molecular groups, depending on the group’s orientation, see Figure 2. In the case of glycine zwitterions, this formalism accounts for the HD-VSFG contributions of the COO^–^ group. The resulting equations involve ratios of the main elements of the molecular hyper-polarizability tensor (β) that were recently experimentally determined^46^ for aliphatic COO^–^ groups connecting to alkyl chains (CH_3_(CH_2_)_*n*_COO^–^), with *n* = 0, 1, 4, 6. Using the approximation that the ratios of the β elements are identical for zwitterionic glycine and propionate (*n* = 1), the following relations hold: Im  and Im , where *C* is a physical constant, and θ is the angle between the surface normal and the symmetry axis of the COO^–^ group, see Figure 2(a). To obtain this result, we assumed the angular distribution of the COO^–^ groups is narrow: Δθ = 0, and that the COO^–^ group can freely rotate around the connecting C–C bond.^46^ In general, the narrow distribution assumption yields sufficiently accurate results for glycine species oriented by direct surfactant interactions or by strong electric fields. In the Supporting Information, we show that in case of field-oriented zwitterions, the thermodynamics-based calculations yield similar  ratios, see Table 2(a,b). Last, we also show that lifting the assumption of free rotation of the COO^–^ group yields similar  trends, see Supporting Information.

**Figure 2:**
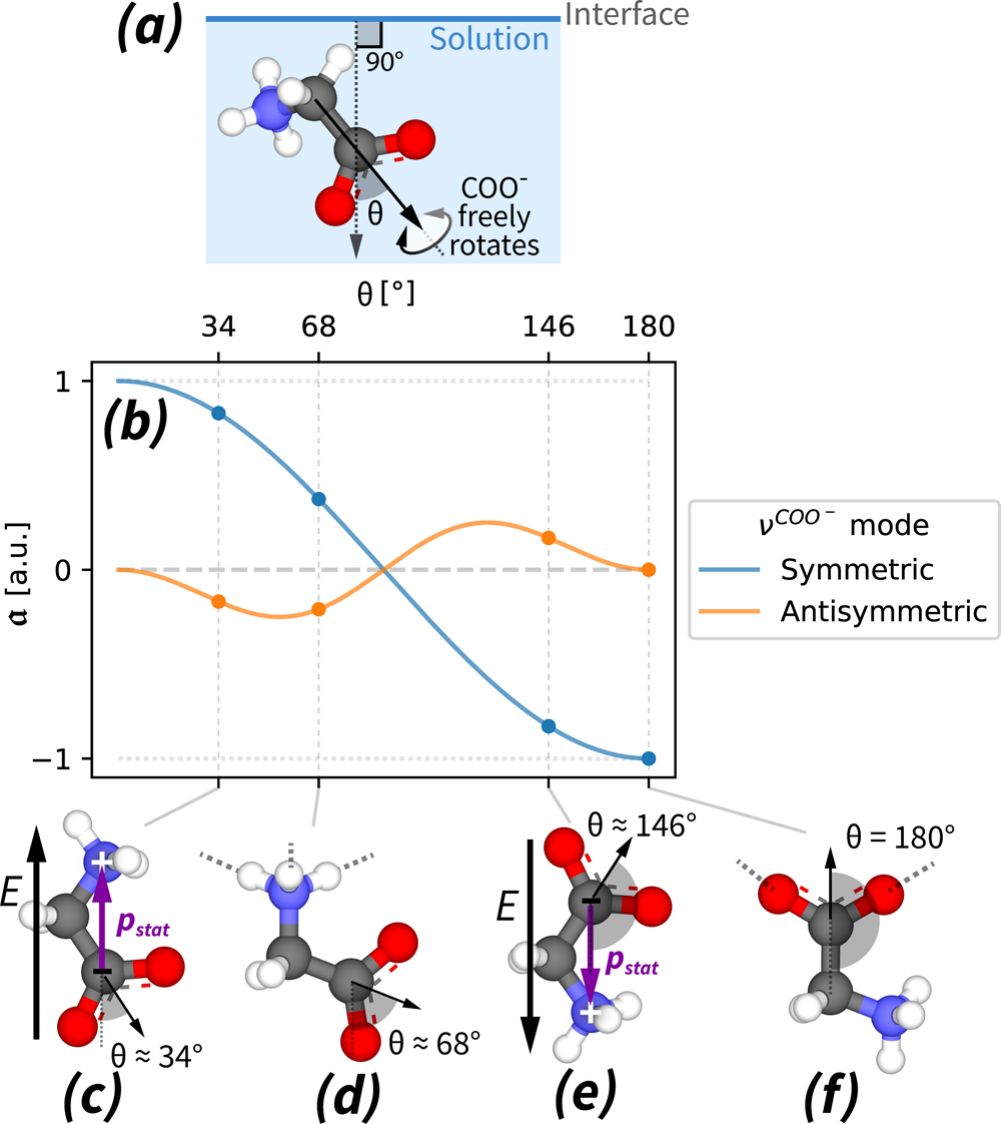
Semiempirical framework connecting the zwitterion’s orientation with the HD-VSFG signals of its COO^–^ group. (a) Definition of the angle θ, as the angle between the surface normal and the symmetry axis of the zwitterion’s COO^–^ group. (b) Relative Im(χ^(2)^) contribution  of the two main carboxylate modes of zwitterionic glycine, derived using the theoretical and experimental results of earlier works.^43−46^ These works use the assumption that the COO^–^ group can freely rotate around the C–C bond. We show  ratios in Table 2. Below: Illustration of zwitterionic glycine molecules, oriented due to (c) the electric field induced by negative surface charges, (d) the interaction of the amine group and the surfactant monolayer, (e) the electric field induced by positive surface charges, and (f) the interaction of the carboxylate group and the surfactant monolayer.

**Table 2:** Comparison of Theoretical Predictions from (a) Figure 2 and (b) a Thermodynamics-Based Model in the Supporting Information; with Experimental Results in D_2_O, from (c) Figure 1(c) and (d) Figure 4^a^

**(a) Theoretical predictions for Figure 2(c–f), Δθ = 0**		
Case	*R*	θ [deg]
*E↑*-oriented	–0.20 ± 0.03	34
NH_3_^+^ interacting	–0.56 ± 0.09	68
*E↓*-oriented	–0.20 ± 0.03	146
COO^–^ interacting	0	180

**(b) Thermodynamics-based theoretical predictions, see Supporting Information**		
Case	*R*	⟨θ⟩ [°] at the interface
*E↑*-oriented	–0.24 ± 0.04	43
*E↓*-oriented	–0.24 ± 0.04	137

**(c) Fit results from Figure 1(c): no added salt**		
Surface coverage	*R*	θ [°]
DS^–^	–0.43 ± 0.01	54 ∓ 1
DTA^+^	–0.18 ± 0.05	149 ± 5
DA^+^	–0.28 ± 0.05	139 ± 5

**(d) Fit results from Figure 4: 1 M added NaCl**		
Surface coverage	*R*	θ [°]
DS^–^	–0.50 ∓ 0.11	61 ± 13
DTA^+^	≈0	∼180
DA^+^	≈0	∼180

a Notation. : Im (χ_SSP_^(2)^) contribution of the vibration .  ratio of COO^–^ Im (χ_SSP_^(2)^) contributions, with  subtracted. θ: COO^–^ angle relative to the surface normal, in case of narrow angular distributions (Δθ = 0). To calculate the above angles, we used a C–C–N angle value^58−61^ of 112°. Experimental Im (χ_SSP_^(2)^) contribution ratios were obtained from the areas of the Gaussians fitted to the observed bands, see fit results in Figures S16 and S15. Experimental errors are calculated using repeated fits of surfactant-covered glycine samples, with an SFG phasing error of ±2°; see Supporting Information. The reported uncertainties in the theoretical *R* ratios are calculated based on the published uncertainties in the β_*aca*_/β_*ccc*_ ratios.^46^.

Using Figure 2(b), we interpret our observations as in Figure 1(c). As noted before, the change of the sign of the surface charge (DS^–^/D(T)A^+^) reverses the direction of the near-surface electric field and, thus, the induced net orientation of the zwitterions (Figure 2(c,e)). Because their orientation flips, so does the sign of the SFG contributions of both the  and the in-plane  vibrations. This kind of orientational flip-flop behavior has been observed before for water^37,47^ ( D)^15,48^ and urea ( D)^49^) molecules.^38^

To better understand the role of electric fields on the HD-VSFG signals, we performed additional experiments with added salts; see Figure 3(a). We find that the addition of Na^+^ and Cl^–^ ions greatly decreases the observed SFG signals. This can be well-explained by ionic screening, which decreases the penetration depth of the electric field induced by the DS^–^ surface covered surface. Similar screening effects have also been observed for interfacial water^47^ and urea^38^ molecules. With the increase of the salt concentration, we additionally observe a small blue-shift of the  signal. This blue-shift can be explained if we consider that at higher ionic strengths, the added ions influence the probed zwitterions’ solvation environment and therefore the vibrational frequency of the COO^–^ group.

**Figure 3:**
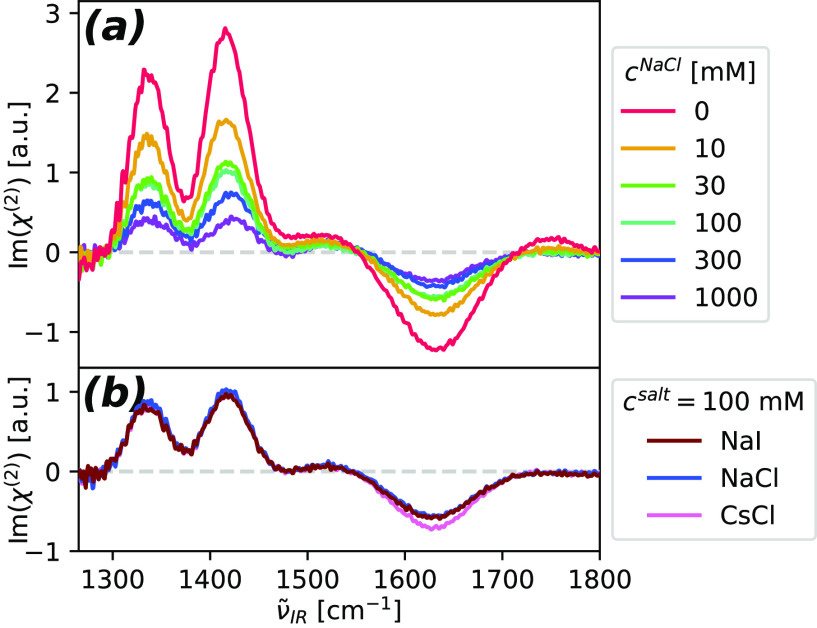
Effect of the addition of salts on the HD-VSFG signals of glycine. (a) HD-VSFG spectra of 2 M glycine in DS^–^-covered H_2_O, with an increasing concentration of added NaCl. The negative feature at 1620 cm^–1^ contains the broad HD-VSFG response of  of zwitterionic glycine as well as that of the  bending mode of H_2_O (ν̃_IR_ = 1643 cm^–1^).^50^ (b) Effect of exchanging salt ions (*c*^salt^ = 100 mM). For both sets of spectra, we subtracted the quadrupolar SFG contribution of neat H_2_O, which does not change with added salt concentration.^47^

In order to investigate ion-specific effects, we repeated the above experiment with different ions, including Na^+^, Cs^+^, Cl^–^, and I^–^, see Figure 3(b) and Figure S14. We find that for all salts the observable HD-VSFG signals match within measurement error. We do not observe new spectral features for any of the solutions, indicating that there is no specific interaction between the added ions and the COO^–^ or the NH_3_^+^ group of zwitterionic glycine. Because we do not detect new spectral features in bulk solutions either (Figure S11), we rule out a significant formation of specific ion-associated species. We therefore infer that in the case of the investigated ions, the screening effect on the HD-VSFG signals of zwitterionic glycine is fully governed by electrostatics.

We repeated the ionic screening experiment for solutions of 1 M glycine with 1 M NaCl added, at the neat D_2_O/air interface and in the presence of DTA^+^, DA^+^, and DS^–^ monolayers; see Figure 4. When compared to Figure 1(c), we find that the observed HD-VSFG signals of the zwitterions are greatly reduced. Overall, the behavior of the  and  bands is consistent with the observations in Figure 3, where the increased ionic screening led to a decrease of the near-surface electric field and thus the net glycine HD-VSFG signal.

**Figure 4:**
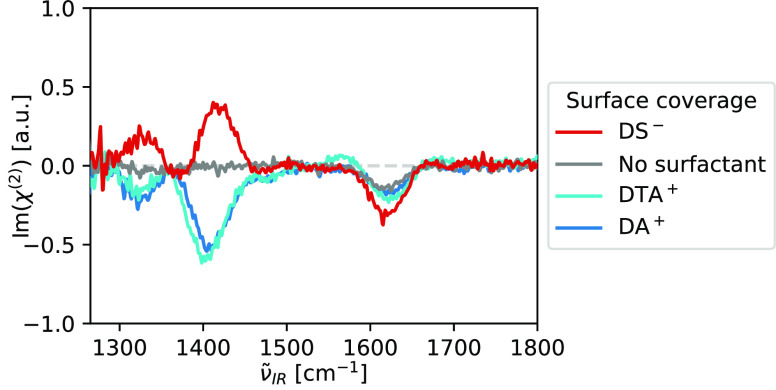
Comparison of HD-VSFG spectra of 1 M glycine + 1 M NaCl solutions, at the neat D_2_O/air interface and in the presence of monolayers of charged surfactants. The addition of Na^+^ and Cl^–^ ions does not change the zwitterion signals at the neat water/air interface, see Figure S12. For display purposes, we subtracted SFG contributions of the corresponding neat/surfactant-covered D_2_O/air interfaces.

The behavior of the  band, however, is not consistent with this picture. In the case of the neat water/air interface, the HD-VSFG contribution of the  band is negative (i.e., ), and the contribution of the  band is zero (i.e., ). This behavior cannot be easily explained by using the theoretical framework presented before. We therefore consider that the observed  values are very similar for the neat water/air interface and for 1 M NaCl solutions covered with D(T)A^+^ monolayers. Due to its apparent insensitivity to electric fields and interfacial properties, we infer that this small negative  does not have a dipolar origin. Nondipolar contributions in HD-VSFG experiments have been experimentally studied in a number of chemical systems: in the case of  of water,^47,51^ ν^OH^ of carboxylic acid dimers,^52^ ν^C=O^ of organic carbonates,^53^ and ν^CH^ of benzene^54^ and its derivatives.^55^ The observed quadrupolar^56,57^ HD-VSFG contributions often carry bulk information: they are generally insensitive to interfacial properties and scale with solute concentration. We indeed observe this; see Figure S8. Since we also find that the small negative  is not changed when adding salts (Figure S12), we tentatively assign this contribution to the quadrupolar HD-VSFG response of the antisymmetric stretching vibration of the zwitterion’s COO^–^ group, i.e., . This also means that zwitterions have no dipolar HD-VSFG contributions at the neat water/air interface, meaning that they do not have a net orientation in the absence of surfactants and electric fields.

To determine the orientation angles of zwitterionic glycine, we use Gaussian peaks to fit all signals in Figures 1(c) and 4 (Figures S16 and S15), and obtain the  amplitudes of the  vibrations. By subtracting the  contribution, we obtain dipolar  ratios, see Table 2(c). We then obtain the average orientation angle θ of the zwitterionic form of glycine near the water surface, by comparing the experimentally obtained ratios with the theoretical values, in case of Δθ = 0. For zwitterions oriented by the electric field, we use the *R* ratios predicted by our thermodynamics-based calculations.

For a solution containing DS^–^ without added salt (Figure 1(c)), we obtain , corresponding to  (Table 2(c)). The obtained  ratio is in between the expected ratio for zwitterions oriented by the electric field: *R*^*E↑*^ = −0.24 (Table 2(b)); and the expected ratio for zwitterions that—via their NH_3_^+^ group—directly interact with the DS^–^ monolayer: ; with  (Figure 2 (d)). Upon adding 1 M NaCl, the obtained  value decreases to −0.50 ∓ 0.11, resulting in a net angle ; see Table 2(d). These values indicate that the orienting effect of the ion-screened electric field decreases compared to the orienting effect of the direct interaction with the DS^–^ surfactant.

In the case of solutions covered with DTA^+^ monolayers, we observed no net response of the  band in Figure 1(c), because the small negative  response cancels the small positive  response. By subtracting  (measured at the neat D_2_O/air interface), we obtain , corresponding to the angle . This observed  ratio is then slightly more positive compared to the predicted response of *E↓*-oriented species, with *R*^*E↓*^ = −0.24 (Table 2(b)). The observed differences can be explained if we consider the emergence of zwitterions with a COO^–^ group that coordinate with the monolayer (, see Figure 2(f)), which would then shift the  ratio to more positive values. Upon the addition of 1 M NaCl, we observe that the  ratio vanishes, which corresponds to an angular value of . This angle indicates that the orientation of the zwitterions is now possibly dominated by a direct interaction between the zwitterion’s COO^–^ group and the DTA^+^ surfactant headgroup. This would mean that orienting effect is only related to the surface angle of the COO^–^moiety, which is optimal at .

In the case of a solution covered with DA^+^ monolayers, we find that the HD-VSFG signals of both the  and  bands increase compared to those in solutions with DTA^+^ monolayers but have a different degree of enhancement: the  contribution roughly doubles while the  contribution increases only by ≈40%; yielding  and . The observed  ratio is more negative than both the  ratio observed with DTA^+^ monolayers present and the predicted *R*^*E↓*^ = −0.24 ± 0.04 ratio in case of field-oriented glycine species. We propose that this might be due to the emergence of zwitterions that have singly coordinated COO^–^ groups, see an illustration in Figure S17. Singly coordinated COO^–^ groups would then have a smaller angle^35^ of , allowing the observed  ratio shift to more negative values. When adding 1 M NaCl, the  ratio reaches approximately zero, similar to the case with DTA^+^ coverage. In case of DA^+^ monolayers, the thus-obtained  value indicates that the dipole-orienting effect of the electric field becomes negligible due to the ionic screening and that the zwitterionic glycine molecules mainly orient due to the specific interaction of their COO^–^ group with the NH_3_^+^ group of the DA^+^ monolayer, see Figure 2(f). The observed *R* value also suggests that we observe a loss of contributions from zwitterions with single-coordinating COO^–^ groups. This can be explained if we consider the electrostatic energy landscape. At 1 M NaCl concentration, the surfactant-induced electric field rapidly decays, and therefore, it mainly interacts with the zwitterions’ COO^–^ moiety. In this case, the most favorable configurations are the ones with the smallest distance between the surfactants’ charged headgroups and the COO^–^ groups charge center. This would then mean that a configuration of zwitterions with  is more favorable than those with single-coordinating COO^–^ groups, where 

In summary, we used heterodyne-detected sum-frequency generation spectroscopy (HD-VSFG) to study the orientational behavior of glycine at the water/air interface in the absence and presence of charged surfactant monolayers. At neutral pH/pD, we found that glycine is predominantly present at the surface in its zwitterionic form. By directly probing the symmetric and antisymmetric stretching vibrations of the zwitterion’s carboxylate group (COO^–^), we find strong evidence that the antisymmetric stretching vibration has a quadrupolar HD-VSFG contribution, which is independent of electric fields. After subtracting this contribution, we can calculate the zwitterions’ COO^–^ group’s net angle of orientation (θ) with respect to the surface normal.

At low ionic strengths, we find the orientation of zwitterionic glycine is predominantly governed by its interaction with the surface-charge-induced electric field, owing to its large dipole moment (16.5 D). At the neat water/air interface, the surface is neutral, and we find that glycine has no preferred orientation. At DTA^+^- and DA^+^-covered surfaces, the observed angles are  and , respectively. These values are very close to the theoretically predicted angle value for field-oriented zwitterions: in the case of narrow angular distributions (Δθ = 0), we predict θ^*E↓*^ = 146°, while our thermodynamics-based calculations predict ⟨θ^*E↓*^⟩ = 137° (calculated at the interface). For a DS^–^-covered surface, we find , indicating at this interface, the orientation of zwitterionic glycine is determined both by the electric field (θ^*E↑*^ = 34°; ⟨θ^*E↑*^⟩ = 43°) and by the direct interaction of the NH_3_^+^ moiety with the SO_3_^–^ group of the surfactant . Overall, the field-induced orientational behavior of zwitterionic glycine shows similarities to the field-induced orientation of water molecules in the diffuse Gouy–Chapman layer.

With enhanced ionic screening present, we observe that the orientation of glycine is increasingly determined by specific interactions of its COO^–^/NH_3_^+^ groups and the positively/negatively charged hydrophilic headgroups of the surfactants. This is indicated by the change of the orientation of glycine: for negatively charged, DS^–^-covered surfaces, we find that the angle  increases toward 68°, while for positively charged, D(T)A^+^-covered surfaces,  increases to ∼180°. To continue our previous analogy: at high ionic strengths, this tendency of zwitterions toward specific interactions could show parallels to the behavior of water molecules in the Stern layer.

We thus find that the near-surface orientation of zwitterionic glycine is determined by both electrostatic and specific interactions. We additionally find that the balance between these interactions and the resulting net orientation both depend on the ionic strength. We anticipate that such information can provide a better understanding of glycine’s behavior near neural synapses and near glycine-specific receptors, where electric fields and specific interactions both play an important role.

## Supporting Information Available

Description of experimental details, data processing approaches and additional measurements.
Transparent Peer Review report available (PDF)
