# Supplementary material for: Orientational Behavior and Vibrational Response of Glycine at Aqueous Interfaces
Source: J Phys Chem Lett. 2024 Feb 15;15(7):2075–81. doi: 10.1021/acs.jpclett.3c02930 (PMC10895693; doi:10.1021/acs.jpclett.3c02930)
Supplement: Supplementary file 1 — jz3c02930_si_001.pdf [file jz3c02930_si_001.pdf]

Orientational Behavior and Vibrational Response of Glycine at Aqueous Interfaces

Balázs Antalicz,* Sanghamitra Sengupta, Aswathi Vilangottunjalil, Jan Versluis, and Huib J. Bakker*

AMOLF, Ultrafast Spectroscopy, Science Park 104, 1098 XG Amsterdam, Netherlands

E-mail: antalicz@amolf.nl; bakker@amolf.nl

Samples and preparation

Chemicals

We obtained purified H_2O from a Simplicity Millipore system, with a resistivity of 18.2 MΩ·cm. We purchased additional chemicals from Sigma Aldrich/Merck, VWR, Deutero and Thermo Fischer. These chemicals were used as received, listed in Table S1. In general, all chemicals were stored in closed containers, in the dark, under nitrogen atmosphere.

Table S1: List of chemical compounds used in our measurements.

Name	Origin	Form	Purity level	Remarks
D_2O	Merck	liquid	99.9 atom% D	-
D_2O	VWR	liquid	99.9 atom% D	-
D_2O	Deutero	liquid	99.9 atom% D	Conductivity $<$ 0.2 mS/m
NaOH	Honeywell/Fluka	1 mol/l solution	-	-
NaOD	Merck	40 wt% solution	-	-
HCl	Merck	37 wt% solution, fuming	-	-
DCl	Merck	35 wt% in D_2O	$>$99. atom% D	-
Glycine	Merck	divided solid	99.%	Suitable for electrophoresis
Glycine	VWR	divided solid	$>$99.6%	Analar Normapur
NaCl	Merck	divided solid	99.5%	-
NaCl	Thermo Fischer	divided solid	99.99%	-
NaI	Merck	divided solid	99.5%	ACS reagent
NaI	Thermo Fischer	divided solid	99.999%	-
CsCl	Merck	divided solid	98.%	For molecular biology
CsCl	Thermo Fischer	divided solid	99.999%	-

In Table S1, we list multiple suppliers for D_2O. This is because we switched suppliers due to fluctuations in availability and pricing. We report, that we find no spectroscopic differences in D_2O from different suppliers, other than slight variations in HOD content.

In case of glycine and salts, we used the first table entry for some H_2O-based measurements (Main Text Figure 3, and SI Figures S6 (b), S9, S10). We then used compounds with enhanced purity for the remaining H_2O-based measurements (SI Figures S6 (a), S7, S8, S11, S12, S13, and S14) and in case of D_2O-based measurements (Main Text Figures 1 and 4, SI Figure S8). We find that our results are repeatable across all purity grades of D_2O used.

Sample preparation methods

We first prepared stock solutions by dissolving solid compounds in the designated solvent. To obtain solutions with the desired concentration, we further diluted and mixed these stock solutions. To calculate the concentration of the stock solutions in mol/liter (M) units, we divide the molar amount of the solute with the volume of the added solvent. In case mixed/diluted solutions, we multiply the concentration of the relevant stock solution by the dilution factor.

To prepare monolayers of surfactants on neat H/D_2O with no added salt, we created solutions with a surfactant concentration of $c = 2\ mM$. We monitor the formation of monolayers using SFG spectroscopy. In case of of Na^+DS^- and DTA^+Br^- solutions, a previous work[1] (see its SI) reported stable $\nu^{CH_{2/3}}$ signals at c= 2 mM. We also report similar findings for DA^+ monolayers, see SI Figure S3.

In case of measurements in H_2O, with added salt and glycine (Main Text Figure 3, SI Figure S13 and S14), we kept the Na^+DS^- concentration at 2 mM. When preparing the stock solutions for these measurements, we additionally used hydrophilic syringe filters (nylon/PES, 0.22 um pore size) to remove possible water-insoluble micro-particles that could otherwise induce precipitation. We find that samples prepared in a such manner were stable for at least 24 hours, much longer than the duration of the SFG measurements.

For D_2O-based samples with added salt and DTA^+ coverage (Main Text Figure 4), when prepared in the above manner, we report months-long solution stability at a surfactant concentration of 2 mM.

For D_2O-based samples with added salt and DS^-/DA^+ coverage (Main Text Figure 4), we used a surfactant concentration of 0.25 mM and otherwise prepared samples in the above manner. We find that such solutions are stable for days, and precipitate if the surfactant concentrations are higher than 0.25 mM. Corroborating monolayer formation, an earlier work reports[2] that with an ionic strength of 0.8 M, SDS has a critical micelle concentration (CMC) of 0.3 mM. Similarly, full monolayer formation of DS^- is additionally confirmed

by a recent SFG study,[3] even at surfactant concentrations as low as 75 μM. Because DA^+ monolayers have a roughly similar critical micelle concentration (CMC)[4] and also exhibit a similar precipitation behaviour, we tentatively conclude that full monolayer formation occurs under the conditions described above.

Steady-state absorption experiments

Experimental methods

Infrared absorption spectra were recorded using the Bruker Vertex 80v Fourier-transform spectrometer with a resolution of 1 cm^{-1}. We placed liquid samples between two CaF_2 windows (25.4 mm diameter, 0.5 mm thick UV-grade, Crystran CAFP25.4-0.5U). These windows were separated by 25-micron fluorinated ethylene propylene (FEP) spacers when executing D_2O-based measurements, or by no spacers in case of H_2O-based measurements. For the latter, sample thickness was indirectly controlled by adjusting the force compressing windows, allowing us to optimize the cell's transmission for the best signal-to-noise ratio.

To prevent ambient absorption of gaseous CO_2 and H_2O vapor, we executed the absorption measurements under nitrogen atmosphere. We started with a background measurement, where no sample cell was placed in the beampath. Next, we recorded and averaged the absorption spectra based on 50 scans of the interferometer. After measurements, sample cells were disassembled, and every optical surface was cleaned with the 'Drop and drag' method:[5] first using water, then with acetone and ethanol.

Numerical methods

Solvent background subtraction and sample thickness normalization

Previously, we described a solvent background subtraction method[6] (see the SI), which allowed us to remove various FTIR contributions of the solvent and the sample cell. We summarize: **1.** First, we characterize CaF_2 windows used in the experiment, and construct the total contribution of a 1 mm window. We subtract this from all measured spectra. **2.** Then, we additionally measure the IR spectra of pure solvents, with and without added H/DCl and NaOH/D; and HOD in case of D_2O. We obtain their component spectra by performing a scaled subtraction on the corresponding combination bands of OH/D stretching + bending modes, and of the OH stretching band in case of HOD.[7] To increase the accuracy

of the subtraction - crucial for thinner, H_2O-based measurements - we scale and subtract all solvent signals in one step, while also accounting for minor fluctuations of the instrument baseline. **3.** We similarly process glycine FTIR spectra, where we additionally subtract H/DCl or NaOH/D component spectra by scaling on a manually selected spectral region between 1500...2100 cm^{-1}, where glycine species do not absorb. Using the scaling constant for neat H/D_2O, we normalize the obtained glycine component spectra with the relative sample thickness. We estimate that this method is approximately ≈5% accurate in case of thicker, D_2O-based samples.

We note, that in Figure S11, we display FTIR spectrum of 1 M glycine + 4 M NaCl solutions in H_2O. In this case, we did not subtract the spectrum of the pure solvent but the spectrum of 4 M NaCl in H_2O. Because zwitterionic glycine is self-buffering with a pH of ≈ 6.2, it is not necessary to subtract signals of H^+/OH^- components.

Lorentzian peak fitting

We performed peak fitting by minimizing the sum of least squares of the difference between glycine component spectra and a set of asymmetric Lorentzian functions.[8] We do not directly re-use the definition from the reference, as changes to the asymmetry parameter also change other parameters. Instead, we developed approximate formulas to keep these parameters constant within a few% range. The definition is as it follows: for a peak height of $\mathfrak{a}$, a center frequency of $\tilde{\nu}_0$, a full-width at half maximum of $\Delta\tilde{\nu}$, and an asymmetry parameter of α, we define the auxiliary parameters s and $d\tilde{\nu}$, as well as the shape function f:

$$s = 1 + \frac{0.78}{1 + 50 \cdot \alpha^{-2}}$$

$$d\tilde{\nu} = \Delta\tilde{\nu} \cdot \left[-\frac{\alpha}{16} + \left(\frac{\alpha}{10}\right)^3 + \left(\frac{\alpha}{9.4}\right)^5 - \left(\frac{\alpha}{7.7}\right)^7 \right]$$

$$f(\mathfrak{a}, \tilde{\nu}_0, \Delta\tilde{\nu}, \alpha) := \frac{\mathfrak{a}}{s} \cdot \frac{1}{1 + 4 \cdot (\frac{\tilde{\nu} + d\tilde{\nu} - \tilde{\nu}_0}{\Delta\tilde{\nu}})^2} \cdot \frac{2}{1 + \exp(\alpha \cdot \frac{\tilde{\nu} + d\tilde{\nu} - \tilde{\nu}_0}{\Delta\tilde{\nu}})}$$

Using this notation, we allow the asymmetry parameter to vary in the $-2 \leq \alpha \leq 2$ region: the peak shape changes from right-shouldered ($\alpha-2$) to symmetric ($\alpha = 0$) to left-shouldered ($\alpha = +2$); while retaining its width, center and amplitude rather accurately.

Sum-frequency generation (SFG) experiments

Experimental methods

We generate 35 fs, 800 nm pulses using a Coherent Legend Elite Duo amplifier, at a repetition rate of 1 kHz, with a pulse energy of 6.5 mJ. We split these pulses to generate broadband mid-infrared (MIR) pulses using an OPA (LightConversion HE-TOPAS) and narrowband 800 nm (VIS) pulses using a home-built pulse-shaper ($\Delta\tilde{\nu}_{FWHM} \approx 25$ cm^{-1}). We then use these in our experimental setup, see Figure S1.

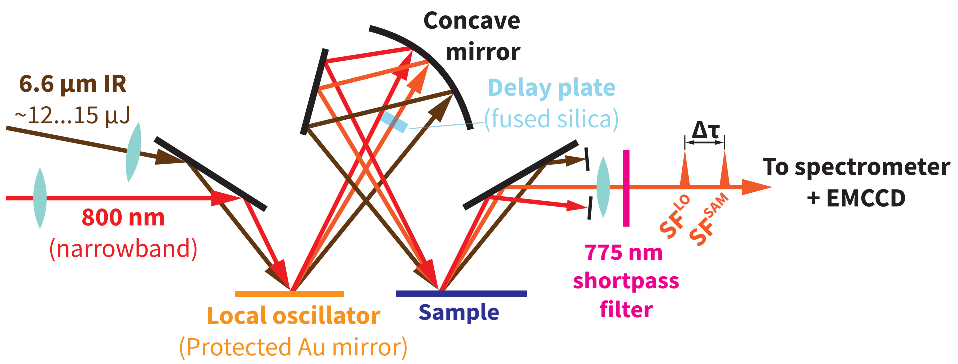

Figure S1: Illustration of the light generation and guidance elements in our heterodyne-detected sum-frequency generation setup. Black lines denote plane mirrors, the arrows denote the direction of laser beams, and cyan shapes denote focusing lenses. SF denotes sum-frequency beams, LO denotes the local oscillator and SAM denotes the sample.

In the experimental setup, we focus both p-polarized MIR and s-polarized VIS pulses onto a local oscillator (Thorlabs PF10-03-M01). We then collect and re-focus the thus generated sum-frequency beam (SF^{LO}) onto the liquid sample in a teflon cell, alongside the MIR and VIS beams. There, the MIR and VIS beams generate a sum-frequency beam again (SF^{SAM}). The VIS/MIR beams are blocked, while the SF^{LO} and SF^{SAM} beams are then re-collimated and analyzed using a spectrometer (Princeton Instruments Acton SpectraPro SP-2300) with a CCD readout (Princeton Instr. Pixis 100). The SF^{LO} and SF^{SAM} beams then interfere on the detector, and produce observable spectral fringes due to the 1 mm fused silica delayplate in the beampath of SF^{LO}. We then record these fringes in the s polarization, and analyze them to extract phase-resolved $\mathrm{Im}(\chi^{(2)}_{SSP})$ spectra.

Signal processing

In Figure S2 we showcase the various steps of signal processing in SFG measurements. First, we record integration-time normalized intensity spectra (I) of a z-cut α-quartz crystal, with normal and with reversed (180° flipped) orientation. This also flips the relative handedness of the sample, which offsets the phase of the generated SFG signals[10] by exactly 180°. We average the normal/reversed quartz signals to obtain a reference spectrum with greatly reduced structural residuals.[1] The steps above are depicted in Figure S2 (a).

Next, we proceed with recording SFG signals of liquid samples, see Figure S2 (b). We employ one-sided Fourier-filtering to extract the corresponding interference fringes:[9]

$$Fringe\, amplitude = Re\{Filtered\, spectrum\} =$$
$$Re\left\{FourierFilter\left(\frac{I^{sample}}{I^{quartz}_{normal} + I^{quartz}_{reverse} + offset}, \tau_{min}, \tau_{max}\right)\right\}$$

Here, we add a small offset to the denominator to the averaged quartz spectrum. This is because at the edges of the recorded spectrum, intensity values are small and noisy; and the inverse of those is extremely noisy. By adding the small offset, we suppress such noise that would otherwise impact data during the Fourier filtering step. During this step, we separate the delayed/oscillatory components using a smooth temporal window composed of two erf functions, centered around $\tau_{min} = \frac{2}{3} \cdot \tau_0$ and $\tau_{max} = \frac{1}{2} \cdot \tau_{Nyquist}$. Here, $\tau_0 \approx 1.75$ ps is the delay between the SF^{LO} and SF^{SAM} pulses induced by the delay plate, and $\tau_{Nyquist}$ is the Nyquist delay.

To obtain $\chi^{(2)}$ spectra, we use the following calculation:

$$\chi^{(2)} = \frac{60 \cdot Filtered\, spectrum^{sample}}{i \cdot Filtered\, spectrum^{quartz}_{normal}}$$

where i is the imaginary unit. This calculation method assumes that quartz does not absorb strongly in this spectral region[11] and thus all its contributions are purely real, hence the usage of i. We note that the multiplication factor of 60 is merely a convention that makes SFG signal amplitudes appear in the range of 1, thus allowing easy handling.

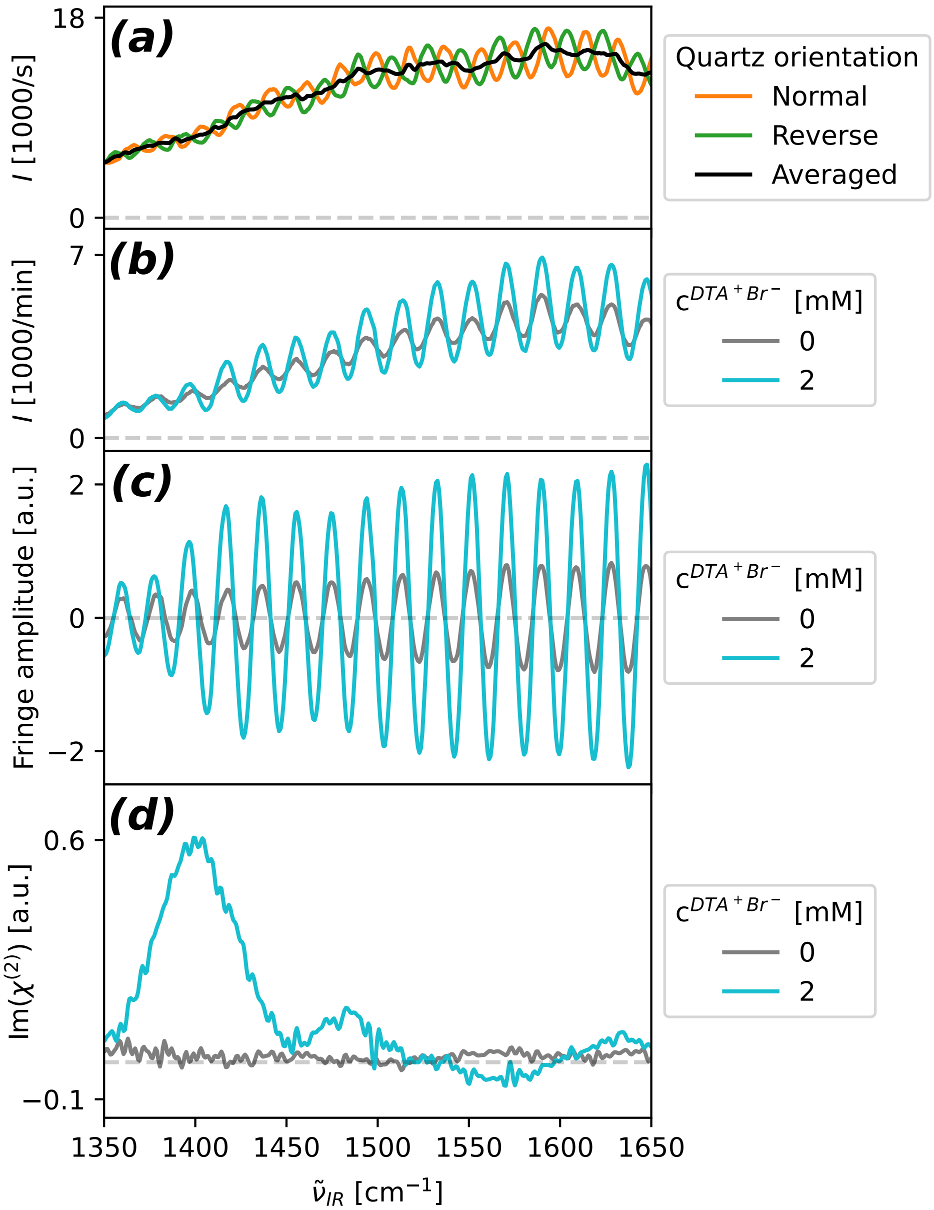

Figure S2: Processing steps in SFG measurements. (a) Integration-time normalized intensity spectra (I) of quartz in normal and reversed position, recorded using a CCD, normalized with integration time. (b) Intensity spectra of D_2O, with and without DTA^+ monolayer present. (c) The real part of recovered signals (i.e. fringes) after Fourier-filtering.[9] (d) Recovered SFG spectra, using a method described earlier.[1]
Note, that around 1400 cm^{-1} where the imaginary $\chi^{(2)}$ contribution of the DTA^+ monolayer is the largest, the recovered fringes of the DTA^+ sample go out of phase compared to the fringes of the D_2O sample.

Additional experimental details

During measurements, the long-term stability of the laser system is a crucial aspect of signal quality. We provide high-quality measurements by frequenting the quartz reference measurements so that typically only 2 samples are recorded between such referencing measurements. Additionally, when we plan to subtract SFG spectra from each other (e.g. at a surfactant-covered surface with and without glycine present), we aim to record these two spectra right after each other, with one quartz reference measurement in-between. This way we have a close and identical reference for the two, allowing for the distinction of subtle differences. In general, we only present measurements in a single figure if those were recorded in a short time window during a measurement day, as to eliminate any possible systematic variations inherent to ultrafast laser systems.

Another experimental consideration is regarding the height of the surface of liquid samples. A different sample and quartz height can cause slight differences in the geometric phase of the SF^{LO} beam and the total accumulated phase of the MIR and VIS beams. We eliminate this by carefully monitoring the vertical position of the SF signals on the CCD, down to a sub-pixel level. We find that this way we can greatly reduce the overall phase uncertainty of our measurements, down to the typical <18° value. To compensate for this, we then apply a manual phase correction whenever necessary. We determine such corrections by analyzing obtained $\chi^{(2)}$ spectra in the 1780...1900 cm^{-1} region, outside of the so-called 'fingerprint region'. Here, samples generally do not absorb and thus we can expect all SFG signals to be purely real. We also confirm the validity of the current approach by monitoring the frequency of the ω^{CH_2} signal of zwitterionic glycine. Due to the Kramers-Kronig relations,[12] the $Re(\chi^{(2)})$ spectrum has a dispersive shape around $Im(\chi^{(2)})$ features. Because an incorrect phasing would intermix the two, such peaks in wrongly phased the $Im(\chi^{(2)})$ would appear to shift in frequency - which is something we do not observe.

Numerical methods

Gaussian peak co-fitting

Similar to FTIR peak fitting, we performed peak fitting by minimizing the sum of least squares of the difference between a set of surfactant/solvent-subtracted SFG spectra (e.g. spectra in Figure 1 (c)) and a set of Gaussian functions. We achieve simultaneous fits of the same peaks by letting individual peak amplitudes vary, while re-using the same width and center parameters. In a single step, a black-box optimizer function (e.g. Python's Numpy's Nelder-Mead or L-BFGS-B methods) takes a set of peak parameters. Using these, we then create the corresponding set of Gaussians with an amplitude of 1. To recover their best-fitting amplitude, we use Numpy's linalg.lstsq function. We then return the least-squares sum to the black-box optimizer; which then iterates through the relevant parameter space until it converges at a solution.

We note, that compared to individual guessing of peak amplitudes, this approach has an increased computational complexity due to the matrix inversion needed. We opted to use it, however, to decrease the number of parameters we need to optimize: for 4 peaks and 4 spectra (e.g. spectra in Figure 1 (c)), we reduce the parameter count from $4 \cdot 4 (amplitudes) + 4 \cdot 2\,(centers\,\&\,widths) = 24$ to $4 \cdot 2\,(centers\,\&\,widths) = 8$. This then allows for an overall faster and more robust parametric convergence.

Auxiliary spectroscopic information

H/D_2O signals under various conditions

We recorded signals of DA^+ monolayers on H_2O, obtained by creating DA^+Br^- solutions of various concentrations, see Figure S3. We find that the CH signals above a 1 mM concentration are stable and that the amplitude of the water signal is the largest of a concentration of 2 mM. This then indicates the formation of stable DA^+ monolayers for the 2 mM concentration case, as we used it in the main text.

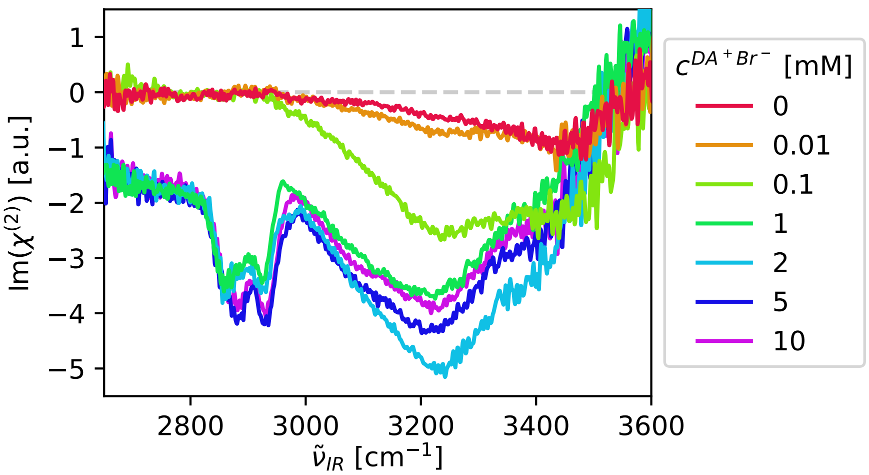

Figure S3: Steady-state SFG spectra of neat water with an increasing concentration of added DAB. These spectra were recorded using another, very similar experimental setup that is optimized for this spectral region.[13]

We additionally recorded SFG signals of neat H_2O, to compare it to 3 M HOD in D_2O, see Figure S4. This is relevant because we work with protonated glycine that gets deuterated due to proton exchange with the solvent. We find that there are no observable δ^{HOD} features[7] around 1450 cm^{-1}.

We also recorded the spectral features of different surfactant monolayers in the fingerprint region, see Figure S5. Here, we used a surfactant concentration of 2 mM. We subtract these features to accurately determine glycine contributions in the main text. Such features were previously reported,[14] but only in a more narrow spectral region.

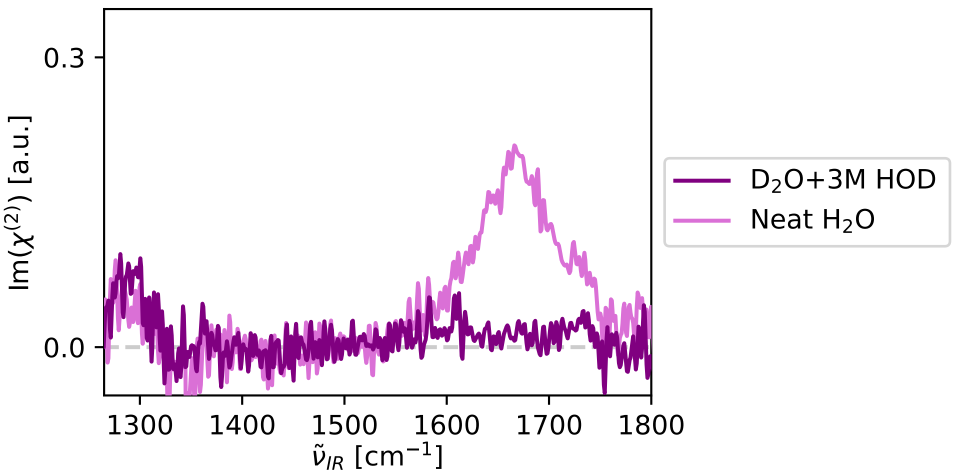

Figure S4: Steady-state SFG spectra of 3 M HOD in D_2O, compared to the SFG spectra of neat H_2O.

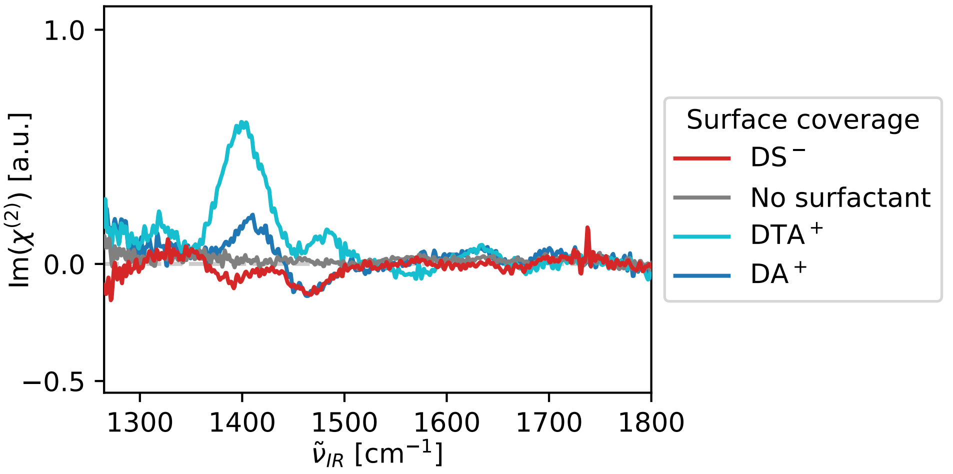

Figure S5: Steady-state SFG spectra of D_2O solutions, at the neat D_2O/air interface, and in the presence of monolayers of charged surfactants ($c^{surfactant} = 2$ mM).

Glycine signals in H_2O

To complete the comparison in the main text, we compare infrared and SFG signals of different glycine species in H_2O, see Figure S6.

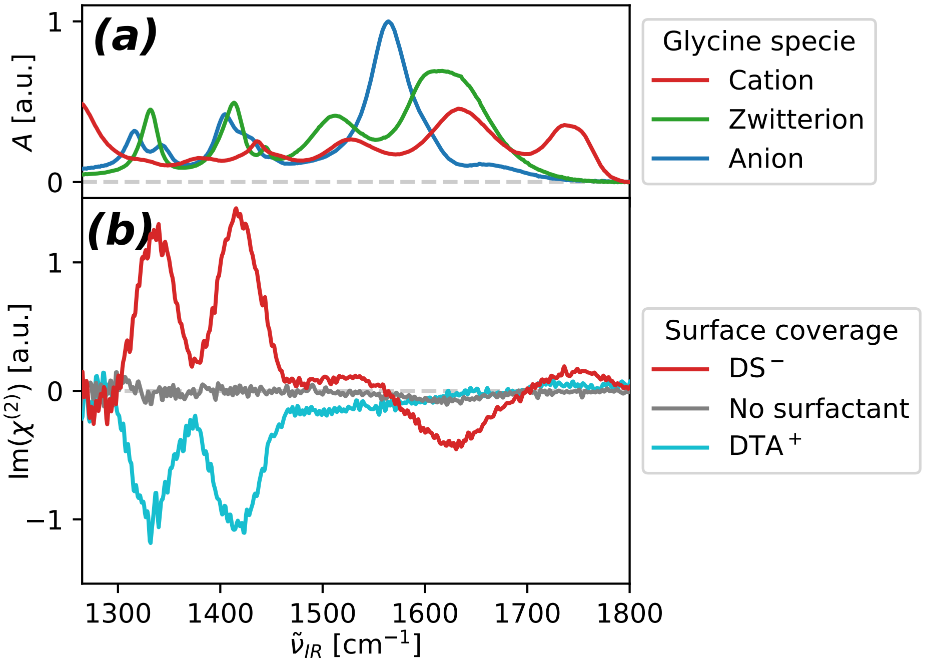

Figure S6: Infrared spectra of different forms of glycine, with solvent/surfactant features subtracted.
(a) Absorption spectra of different species recorded at acidic/neutral/basic H_2O solutions, and are normalized with solute concentration ($c = 550$ mM) and sample thickness. Spectra of anionic and cationic glycine species have a slightly elevated baseline at lower frequencies. (b) Steady-state SFG spectra of 1 M glycine solutions at neutral pH, with different surfactants added. For display purposes, we separately recorded and subtracted SFG spectra of the neat surfactant solutions.

Decomposing steady-state absorption spectra of different glycine species

To decompose the observed spectra to individual peaks in Figure S7, we used the peak fitting methods we described before. We display the fit results in D_2O in Table S2, and in H_2O in Table S3.

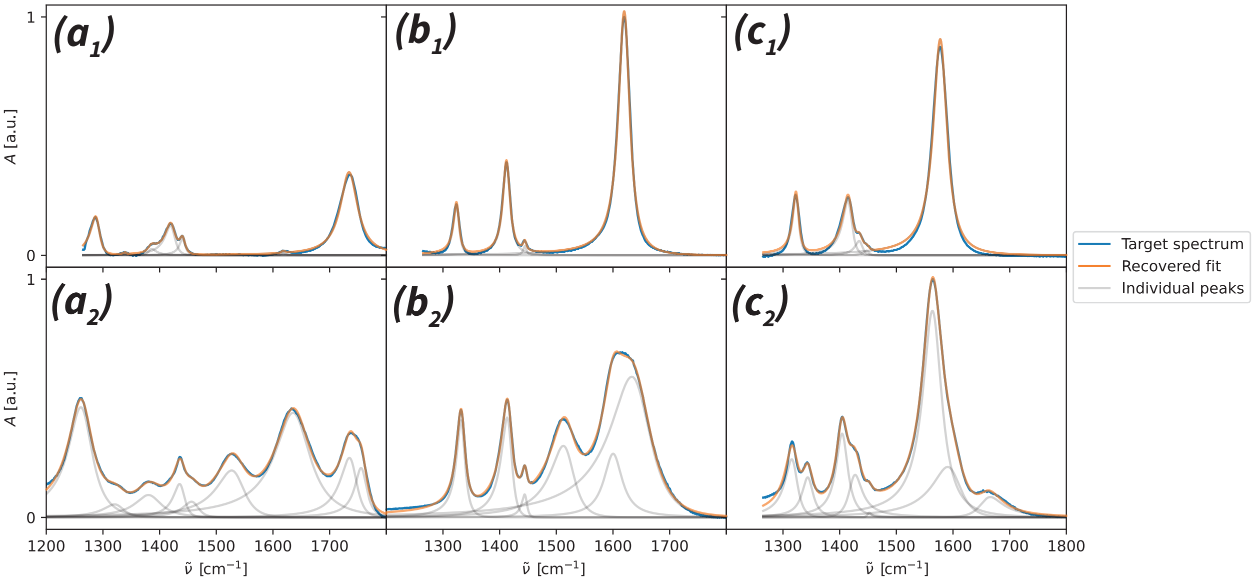

Figure S7: Peak fitting results of glycine in D_2O (top row) and in H_2O (bottom row), using steady-state absorption spectra from main text Figure 1 (b) and SI Figure S6 (a). Left to right: ($a_{1,2}$) cationic, ($b_{1,2}$) zwitterionic, ($c_{1,2}$) anionic glycine species. We display fit parameters in Table S2 for deuteron-exchanged glycine species, and in Table S3 for glycine species in H_2O.

Table S2: Peak fit parameters from Figure S7, for glycine species in D_2O. For notation, see the section 'Peak fitting'.

Specie	$\tilde{\nu}_0$ [cm^{-1}]	$\mathfrak{a}$ (rel. amplitude)	$\Delta\tilde{\nu}$ [cm^{-1}]	α (asymmetry)
cation	1286.9	0.16	19.8	2.0$^{\#}$
	1338.9*	0.01	5.7	2.0$^{\#}$
	1386.2	0.03	16.8	-2.0$^{\#}$
	1419.1*	0.12	21.8	2.0$^{\#}$
	1440.2	0.07	12.6	1.0
	1617.5$^{\bullet}$	0.01	8.3	-2.0$^{\#}$
	1734.3	0.35	37.1	0.1
zwitterion	1323.7	0.22	12.0	0.8
	1412.0	0.39	15.2	0.2
	1444.4	0.04	6.7	0.1
	1619.8	1.02	24.3	0.4
anion	1322.7	0.26	12.8	0.9
	1414.3	0.23	20.2	2.0$^{\#}$
	1434.3*	0.06	16.1	2.0$^{\#}$
	1447.6*	0.02	11.3	2.0$^{\#}$
	1577.3	0.91	29.3	0.3

*Less accurate peak parameters due to small peak amplitude.

$^{\bullet}$Possible zwitterion contribution.

$^{\#}$At maximum limit: $-2 \leq \alpha \leq 2$.

Table S3: Peak fit parameters from Figure S7, for glycine species in H_2O. For notation, see the section 'Peak fitting'.

Specie	$\tilde{\nu}_0$ [cm^{-1}]	$\mathfrak{a}$ (rel. amplitude)	$\Delta\tilde{\nu}$ [cm^{-1}]	α (asymmetry)
cation	1260.9	$0.46^{\bullet}$	52.0	0.9
	1320.5	$0.05^{\bullet}$	45.0	$2.0^{\#}$
	1380.4	$0.09^{\bullet}$	56.4	0.6
	1435.2	$0.14^{\bullet}$	24.7	$2.0^{\#}$
	1456.3	0.07	34.2	$2.0^{\#}$
	1527.1	0.20	59.9	$2.0^{\#}$
	1635.4^{*}	0.44	75.7	0.6
	1734.9	0.25	35.7	$2.0^{\#}$
	1755.5	0.21	25.6	1.0
zwitterion	1331.8	0.41	18.4	1.0
	1413.2	0.42	22.9	$2.0^{\#}$
	1444.1	0.10	11.4	$2.0^{\#}$
	1512.4	0.30	51.2	1.9
	1600.3^{*}	0.27	39.3	0.1
	1633.1^{*}	0.59	86.6	1.6
anion	1315.4	$0.24^{\bullet}$	26.9	1.6
	1343.6	$0.17^{\bullet}$	23.8	0.4
	1404.3	$0.35^{\bullet}$	25.2	0.5
	1427.3	$0.18^{\bullet}$	28.6	-1.3
	1452.2	0.02	8.9	$-2.0^{\#}$
	1563.7^{*}	0.87	40.6	0.4
	1590.6^{*}	0.21	61.9	$2.0^{\#}$
	1665.9^{*}	0.09	50.8	$-2.0^{\#}$

*Fit parameters are less accurate due to the overlap of subtracted δ^{H_2O}.
$^{\bullet}$Less accurate amplitude value due to slightly elevated baseline.
$^{\#}$At maximum limit: $-2 \leq \alpha \leq 2$.

Concentration effects on glycine SFG signals

In the main text, we refer to the concentration dependence of the observed glycine SFG signals. In Figures S8, S9 and S10, we show how adding more glycine does indeed concentration-linearly increase the observable glycine signals, regardless of surface coverage.

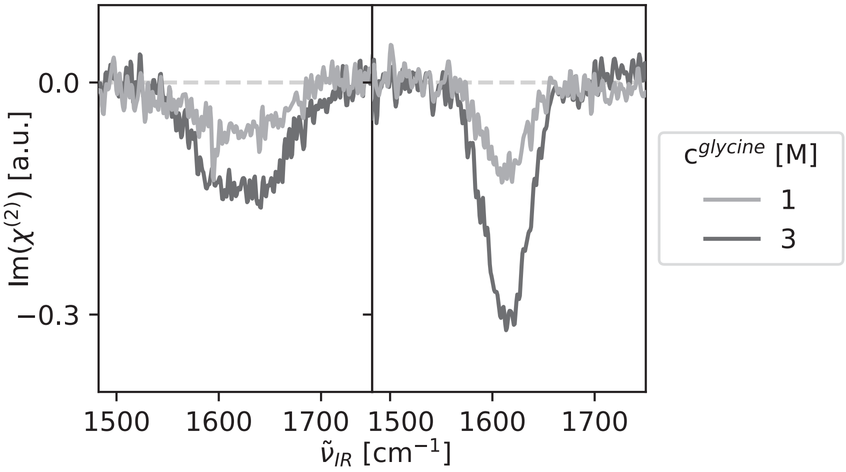

Figure S8: Concentration effects on the steady-state SFG spectra of glycine in neat H/D_2O. For display purposes, we subtracted the SFG spectra of the neat solvents.

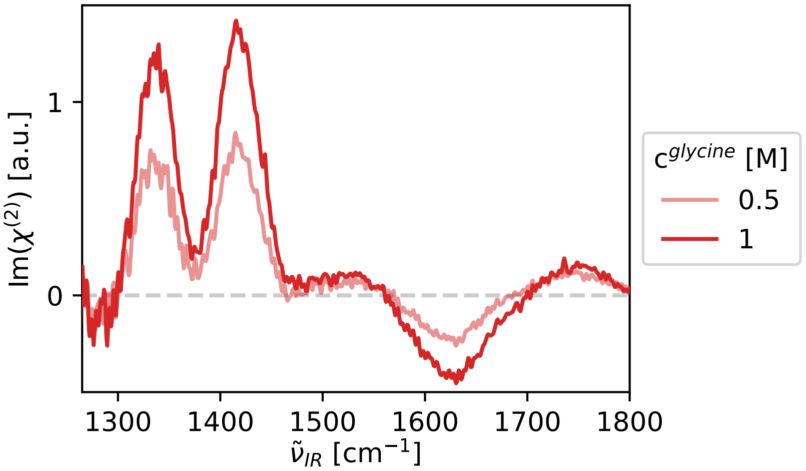

Figure S9: Concentration effects on the steady-state SFG spectra of in H_2O, with 2 mM of SDS added. For display purposes, we subtracted the SFG spectrum of the SDS-covered water surface.

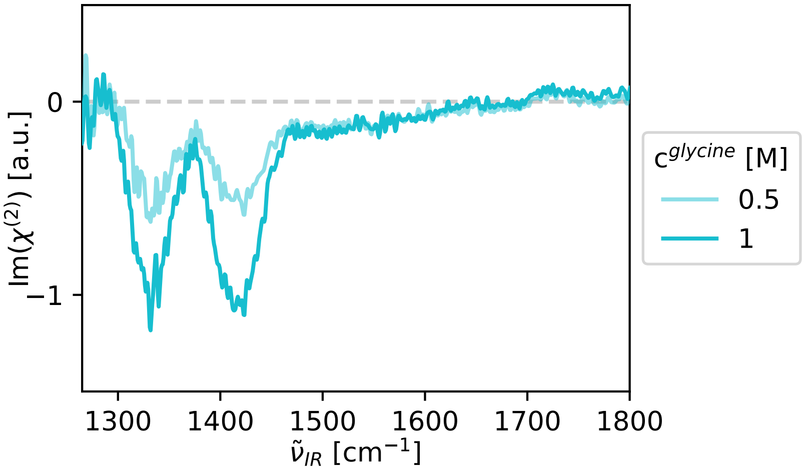

Figure S10: Concentration effects on the steady-state SFG spectra of glycine in H_2O, with 2 mM of DTAB added. For display purposes, we subtracted the SFG spectrum of the DTAB-covered water surface.

Salt effects on the zwitterionic glycine signals

We recorded steady-state infrared absorption spectra of zwitterionic glycine, with and without added NaCl, see Figure S11. Here, we showcase that no new spectral features emerge due to possible salt-carboxylate interaction.[15] We additionally display similar results for SFG spectra, see Figures S12 and S13 and S14.

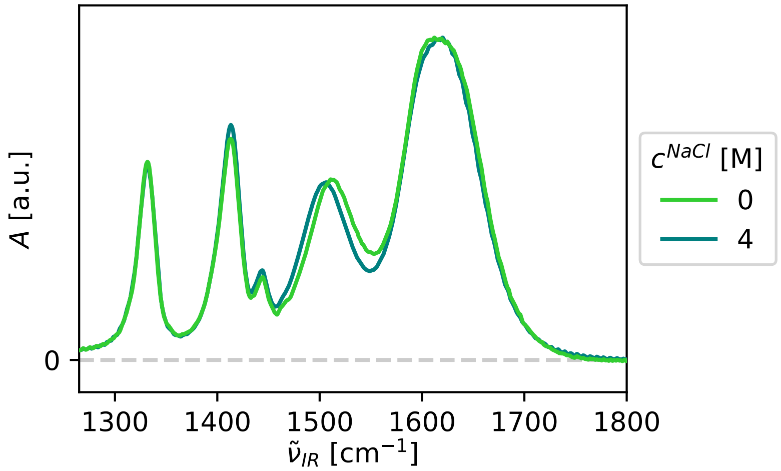

Figure S11: Comparison of solvent-subtracted steady-state infrared absorption spectra of 1M glycine in H_2O, without and with added NaCl. Spectra are normalised with respect to their maximal amplitude.

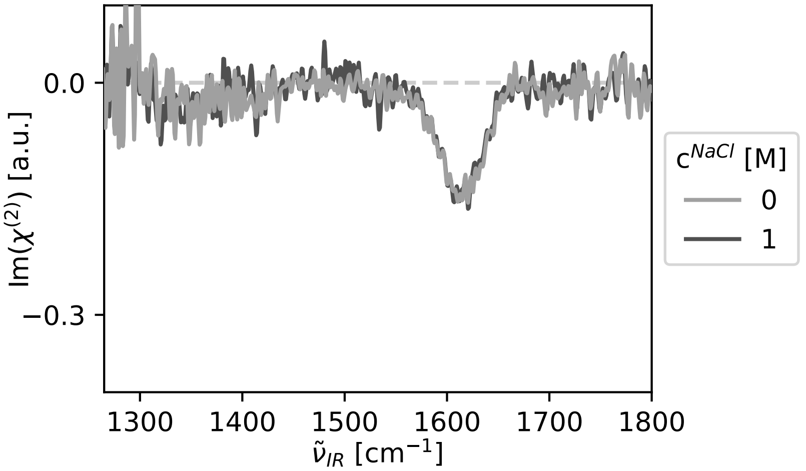

Figure S12: Steady-state SFG spectra of 1 M glycine in D_2O, with and without added NaCl. For display purposes, we subtracted the SFG spectrum of neat D_2O.

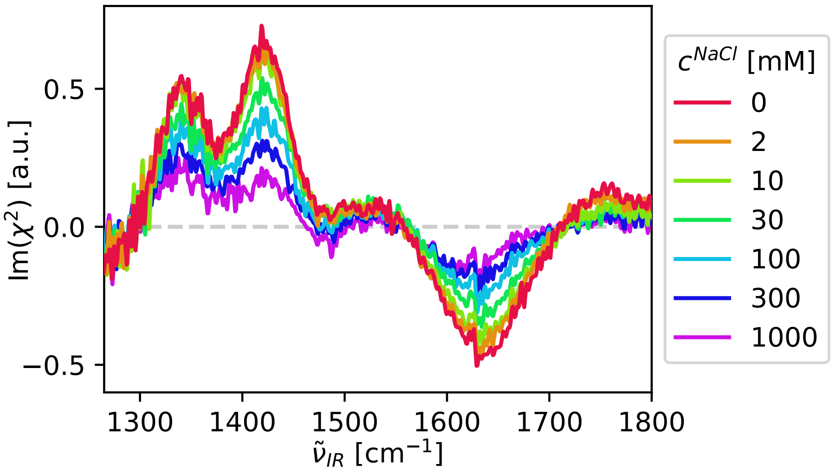

Figure S13: Reproduction of main text Figure 3 (a), using 1 M of ultra-pure glycine and ultra-pure NaCl. For display purposes, we subtracted the quadrupolar SFG contribution of neat H_2O, which does not change with added salt concentration.

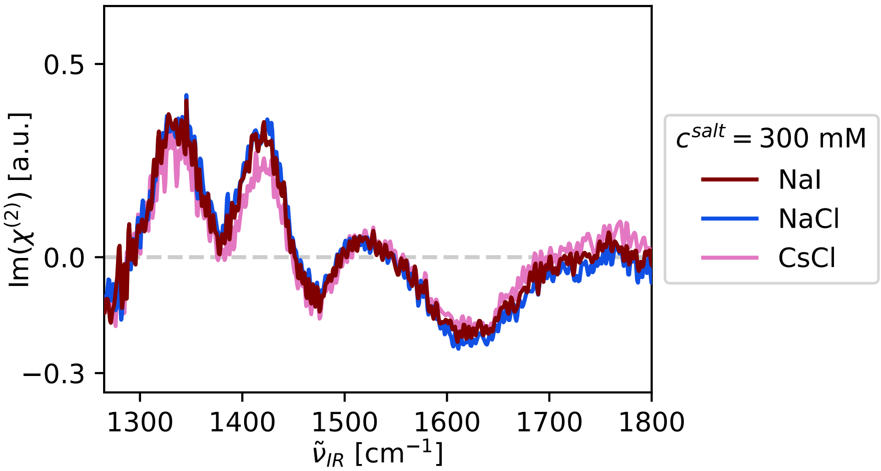

Figure S14: Reproduction of of main text Figure 3 (b), using 1 M ultra-pure glycine and 300 mM ultra-pure salts. For display purposes, we subtracted the quadrupolar SFG contribution of neat H_2O.

SFG peak fitting for D_2O-based measurements

In Figures S15 and S16, we performed Gaussian peak co-fit using the methods described earlier. For error calculation of retrieved amplitudes, we additionally fit SFG signals with added a $\pm 2°$ phase error to the measurements of glycine in presence of surfactants. We then calculate the obtained errors: $\Delta\mathfrak{a} = \frac{1}{2} \cdot (\mathfrak{a}^{(+2°)} - \mathfrak{a}^{(-2°)})$; and similarly $\Delta\theta = \frac{1}{2} \cdot (\theta\{\mathfrak{a}^{(+2°)}\} - \theta\{\mathfrak{a}^{(-2°)}\})$.

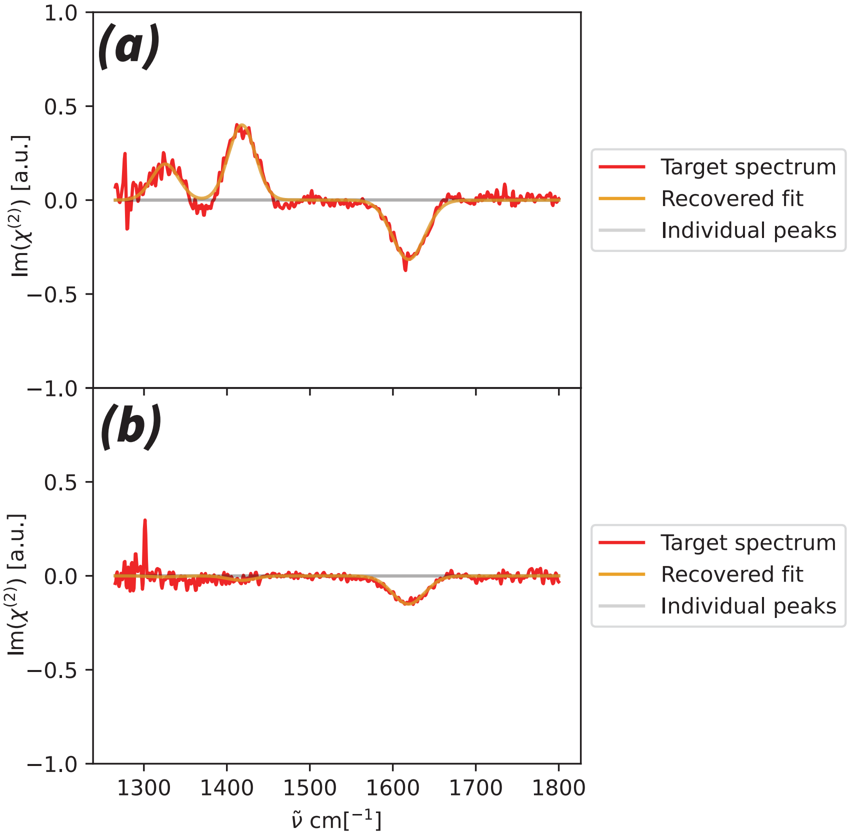

Figure S15: Signal fit from main text Figure 4: Surfactant-subtracted glycine signals (a) with a DS^- monolayer + 1 M NaCl present, (b) at the neat water/air interface + 1 M NaCl present.

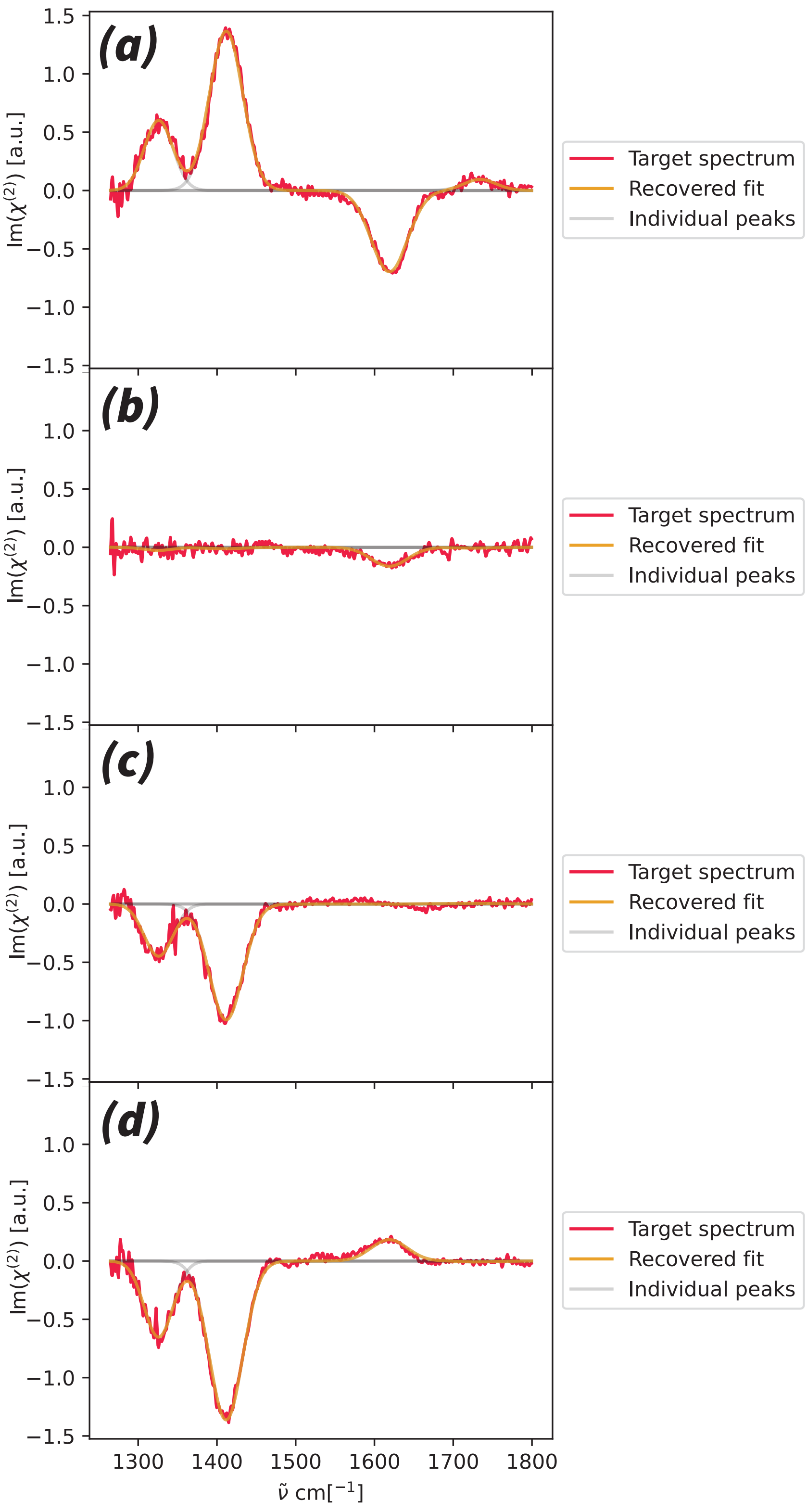

Figure S16: Signal fit from main text Figure 1 (c): Surfactant-subtracted glycine signals (a) with a DS^{-} monolayer present, (b) at the neat water/air interface, (c) with a DTA^{+} monolayer present, and (d) with a DA^{+} monolayer present.

Additional illustrations

Glycine species with alternative orientations

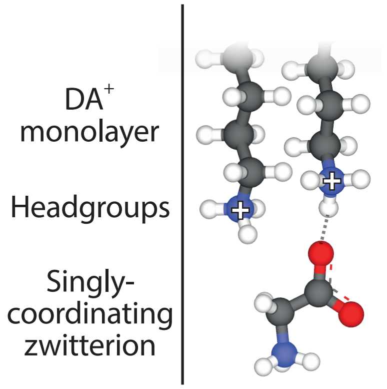

Figure S17: Possible configuration for singly-coordinating glycine species, yielding $\theta \sim 120°$.

Theoretical derivations

$\nu_{s/as}^{COO^-}$ SFG contributions of zwitterionic glycine without freely rotation COO^- moieties

Previous works[16,17] established a framework connecting the observable $\chi_{eff}^{(2)}$ contributions of moieties with C_{2v} symmetry groups, such as COO^- groups. For ensembles of molecules with a narrow angular distribution, where the average surface normal - C_{2v} symmetric axis is θ and the COO^- group is assumed to rotate freely, a later work[18] calculated the following:

$$Im(\chi_{SSP}^{(2)})\{\nu_s^{CCO^-}\} \propto +\frac{1}{4}\cdot(\beta_{aac}+\beta_{bbc}+2\cdot\beta_{ccc})\cdot\cos\theta+\frac{1}{4}(\beta_{aac}+\beta_{bbc}-2\cdot\beta_{ccc})\cdot\cos^3\theta$$
$$Im(\chi_{SSP}^{(2)})\{\nu_{as}^{CCO^-}\} \propto -\frac{1}{2}\cdot\beta_{aca}\cdot(\cos\theta-\cos^3\theta)$$

Previously, β_{aca}/β_{ccc} and $(\beta_{aac}+\beta_{bbc}):\beta_{ccc}$ were experimentally determined for several carboxylates.[19] If we approximate that the result for acetate ion approximately matches the result for zwitterionic glycine, we can now substitute $\beta_{aca}/\beta_{ccc}=1.3$ and $(\beta_{aac}+\beta_{bbc})/\beta_{ccc}=2$, to get the formulas in the main text:

$$Im(\chi_{SSP}^{(2)})\{\nu_s^{CCO^-}\} \propto +\cos\theta$$
$$Im(\chi_{SSP}^{(2)})\{\nu_{as}^{CCO^-}\} \propto -0.65\cdot(\cos\theta-\cos^3\theta)$$

We note, that such an assumption - i.e. the free rotation of COO^- groups in aqueous glycine - might not always be justified. A previous MD simulation[20] suggested that the COO^- group might have a preferential orientation, in the C-C-N plane; where the width of

the angular distribution is $\approx 50°$. This distribution is an in-between case for the fully free COO^- rotation, and between an in-plane locked one. To investigate the SFG contributions of the in-plane locked COO^- group as an extreme limit, we derive the corresponding formulas below.

Based on earlier works,[16,17] we find that for a carboxylate group with the Euler rotations of (ϕ, θ, χ) (check reference for conventions), the observable SFG contribution is the following:

$$\begin{aligned} Im(\chi^{(2)}_{SSP})\{\nu_s^{CCO^-}\} \propto \beta_{aac} \cdot [& +\frac{1}{2} \cdot \cos\theta \cdot (1+\cos 2\chi \cdot \cos 2\phi) \\ & -\frac{1}{16} \cdot (\cos\theta - \cos 3\theta) \cdot (1+\cos 2\chi) \cdot (1+\cos 2\phi) \\ & -\frac{1}{4} \cdot (1+\cos 2\theta) \cdot \sin 2\chi \cdot \sin 2\phi \quad] \\ + \beta_{bbc} \cdot [& +\frac{1}{2} \cdot \cos\theta \cdot (1-\cos 2\chi \cdot \cos 2\phi) \\ & -\frac{1}{16} \cdot (\cos\theta - \cos 3\theta) \cdot (1+\cos 2\chi) \cdot (1-\cos 2\phi) \\ & +\frac{1}{4} \cdot (1+\cos 2\theta) \cdot \sin 2\chi \cdot \sin 2\phi \quad] \\ + \beta_{ccc} \cdot [& +\frac{1}{8} \cdot (\cos\theta - \cos 3\theta) \cdot (1+\cos 2\chi) \quad] \end{aligned}$$

$$\begin{aligned} Im(\chi^{(2)}_{SSP})\{\nu_{as}^{CCO^-}\} \propto \beta_{aca} \cdot [& -\frac{1}{16} \cdot (\cos\theta - \cos 3\theta) \cdot (1+\cos 2\chi) \cdot (1+\cos 2\phi) \\ & +\frac{1}{8} \cdot (1-\cos 2\theta) \cdot \sin 2\chi \cdot \sin 2\phi \quad] \\ + \beta_{caa} \cdot [& -\frac{1}{16} \cdot (\cos\theta - \cos 3\theta) \cdot (1+\cos 2\chi) \cdot (1+\cos 2\phi) \\ & +\frac{1}{8} \cdot (1-\cos 2\theta) \cdot \sin 2\chi \cdot \sin 2\phi \quad] \end{aligned}$$

If COO^- group is fixed in the C-C-N plane, then ϕ=0. Assuming that molecules of an ensemble have no well-defined distribution around the surface normal, we average for $0 \leq \chi \leq 2 \cdot \pi$, and obtain:

$$Im(\chi^{(2)}_{SSP})\{\nu^{CCO^-}_{s}\} \propto \beta_{aac} \cdot [+\frac{1}{2} \cdot \cos\theta - \frac{1}{8} \cdot (\cos\theta - \cos 3\theta)] + \beta_{bbc} \cdot [+\frac{1}{2} \cdot \cos\theta] + \beta_{ccc} \cdot [+\frac{1}{8} \cdot (\cos\theta - \cos 3\theta)]$$

$$Im(\chi^{(2)}_{SSP})\{\nu^{CCO^-}_{as}\} \propto \beta_{aca} \cdot [-\frac{1}{8} \cdot (\cos\theta - \cos 3\theta)] + \beta_{caa} \cdot [-\frac{1}{8} \cdot (\cos\theta - \cos 3\theta)]$$

Using the substitutions[18] $\beta_{aca} = \beta_{caa}$ and $(\beta_{aac} + \beta_{bbc})/\beta_{ccc} = 2$, we get:

$$Im(\chi^{(2)}_{SSP})\{\nu^{CCO^-}_{s}\} \propto \beta_{ccc} \cdot [+\frac{9}{8} \cdot \cos\theta - \frac{1}{8} \cdot \cos 3\theta] + \beta_{aac} \cdot [-\frac{1}{8} \cdot (\cos\theta - \cos 3\theta)]$$

$$Im(\chi^{(2)}_{SSP})\{\nu^{CCO^-}_{as}\} \propto \beta_{aca} \cdot [-\frac{1}{4} \cdot (\cos\theta - \cos 3\theta)]$$

Using the experimentally derived substitutions[19] $\beta_{aca}/\beta_{ccc} = 1.3$, while also neglecting the small $\frac{\beta_{aac}}{8} \cdot (\cos\theta - \cos 3\theta)$ term, we obtain the following:

$$Im(\chi^{(2)}_{SSP})\{\nu^{CCO^-}_{s}\} \propto +0.125 \cdot (9 \cdot \cos\theta - \cos 3\theta)$$

$$Im(\chi^{(2)}_{SSP})\{\nu^{CCO^-}_{as}\} \propto -0.325 \cdot (\cos\theta - \cos 3\theta)$$

We then illustrate the results in Figure S18. Note, that within the approximate range of

$0 \leq \beta_{aac}/\beta_{ccc} \leq 3$, the solution for $Im(\chi^{(2)}_{SSP})\{\nu_s^{CCO^-}\}(\theta)$ remains monotonously decreasing in the $0 \leq \theta \leq 180°$ region. This then suggests that the exact β_{aac}/β_{ccc} ratio is not critical, because it can be varied within a wide range of values without changing the trend-wise behaviour of the observable SFG signal.

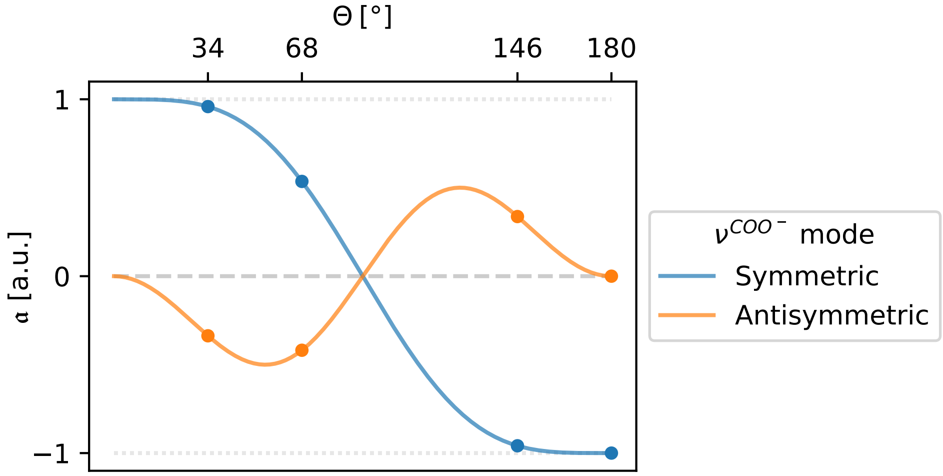

Figure S18: Relative $\mathrm{Im}(\chi^{(2)})$ contribution of the two main carboxylate modes of zwitterionic glycine, assuming that the COO^- group is locked in-plane. Here, we neglect the small β_{aac} term in the derivation above.

$\nu_{s/as}^{COO^-}$ SFG contributions for field-oriented zwitterions: a thermodynamic approach

Using thermodynamic calculations, we obtain the $\mathfrak{a}_{as}/\mathfrak{a}_s$ ratio for the surfactant monolayers in the main text. First, we consider the electric field (E) in the direct vicinity of the interface:

$$E = \frac{\sigma}{\varepsilon \cdot \varepsilon_0} \tag{1}$$

where σ marks the charge density of the surfactants, and $\varepsilon = 78.4$ is the relative dielectric permittivity of water;[21] while ε_0 is the dielectric permittivity of vacuum. This electric field then decays with the Debye length of λ_D (dependent on the ionic strength); to yield

$$E(z) = E(z=0) \cdot e^{-\frac{z}{\lambda_D}} \tag{2}$$

In this field, glycine zwitterions are aligned due to the torque exerted on their static dipole (p_{stat}) by the electric field. This result in the following potential:

$$U = -\overrightarrow{E} \cdot \overrightarrow{p_{stat}} = -U_0 \cdot \cos\alpha \tag{3}$$

where $U_0 = \left|\overrightarrow{E}\right| \cdot |\overrightarrow{p_{stat}}| \cdot sign(\sigma)$, and α marks the angle between the two vectors. Using Poisson-Boltzmann distribution, we can now calculate the net angle:

$$<\alpha>= \frac{\int_0^\pi e^{\frac{U_0 \cdot \cos\alpha}{k_B \cdot T}} \cdot f(\alpha) \cdot \alpha \cdot d\alpha}{\int_0^\pi e^{\frac{U_0 \cdot \cos\alpha}{k_B \cdot T}} \cdot f(\alpha) \cdot d\alpha} \tag{4}$$

where $f(\alpha) = 2 \cdot \pi \cdot \sin\alpha$ represents the density of available states.

To obtain $\mathfrak{a}_{s/as}$ values, however, we need θ, not α values. Here, we consider that the angle between the symmetry axis of the COO^- group is fixed: $\delta = 34°$, see main text. As long as α is fixed, the zwitterion can freely rotate around its dipole, without further energy penalty. We thus define the angle β, as the one between the plane stretched by the $\overrightarrow{E_0}$ and

$\overrightarrow{p_{stat}}$ vectors, and the C-C-N plane of the zwitterion. Using this definition, we can calculate θ for any given α and β. To do that, let's consider the following definitions:

$$\overrightarrow{p_{stat}} := (0, \sin\alpha, \cos\alpha) \tag{5}$$

where the z axis is perpendicular to the surface, and points towards the bulk of the solution. We then calculate the orientation of the symmetry axis $\overrightarrow{s^{COO^-}}$:

$$\overrightarrow{s^{COO^-}} := \underline{\underline{R_x}}(-\alpha) \cdot (\sin\delta \cdot \sin\beta, \sin\delta \cdot \cos\beta, \cos\delta) \tag{6}$$

where $\underline{\underline{R_x}}$ is the rotation matrix around the x axis. By substituting its well-known value, we can calculate the corresponding values of $\overrightarrow{s^{COO^-}}$ in the laboratory framework. Per the definition of θ, we only need its z-component:

$$\cos\theta = z\left(\overrightarrow{s^{COO^-}}\right) = -\sin\alpha \cdot \sin\delta \cdot \cos\beta + \cos\alpha \cdot \cos\delta \tag{7}$$

This now allows us to calculate $< \mathfrak{a}_{s/as} >$ values for any given α:

$$< \mathfrak{a}_s > (\alpha) =< \cos\theta >= \frac{\int_0^{2\cdot\pi} \cos\theta \cdot d\beta}{2\cdot\pi} = \frac{\int_0^{2\cdot\pi} (-\sin\alpha \cdot \sin\delta \cdot \cos\beta + \cos\alpha \cdot \cos\delta) \cdot d\beta}{2\cdot\pi} \tag{8}$$

$$< \mathfrak{a}_{as} > (\alpha) = -0.65 \cdot < \cos\theta - \cos^3\theta >= \frac{\int_0^{2\cdot\pi} 0.65 \cdot (\cos\theta - \cos^3\theta) \cdot d\beta}{2\cdot\pi} \tag{9}$$

Such calculations can be done numerically.

We can now calculate $< \mathfrak{a}_{s/as} >$ values for any given electric field:

$$< \mathfrak{a}_s > \{E\} = \frac{\int_0^\pi e^{\frac{U_0\{E\}\cdot\cos\alpha}{k_B\cdot T}} \cdot F(\alpha) \cdot < \mathfrak{a}_s > (\alpha) \cdot d\alpha}{\int_0^\pi e^{\frac{U_0\{E\}\cdot\cos\alpha}{k_B\cdot T}} \cdot F(\alpha) \cdot d\alpha} \tag{10}$$

$$< \mathfrak{a}_{as} > \{E\} = \frac{\int_0^\pi e^{\frac{U_0\{E\}\cdot\cos\alpha}{k_B\cdot T}} \cdot F(\alpha) \cdot < \mathfrak{a}_{as} > (\alpha) \cdot d\alpha}{\int_0^\pi e^{\frac{U_0\{E\}\cdot\cos\alpha}{k_B\cdot T}} \cdot F(\alpha) \cdot d\alpha} \tag{11}$$

where $F(\alpha) = 2 \cdot \pi \cdot \sin\alpha$.

Using this, we calculate $\mathfrak{a}_{s/as}$ values for any given electric field distribution, by sampling the electric field at a depth z:

$$\mathfrak{a}_{s/as} = \int_0^\infty < \mathfrak{a}_{s/as} > \{E(z)\} \, dz \tag{12}$$

For exponentially decaying $E(z)$ profiles, that start match the conditions above (i.e. $E(z=0) = 0.46\ \frac{GV}{m}$), we obtain that $\mathfrak{a}_{as}/\mathfrak{a}_s = -0.24$. This value is independent of the Debye lengths, and is also very close to the $\mathfrak{a}_{as}/\mathfrak{a}_s = -0.20$ value, predicted in case of narrow angular distributions.

References

(1) Moll, C. J.; Versluis, J.; Bakker, H. J. Direct Evidence for a Surface and Bulk Specific Response in the Sum-Frequency Generation Spectrum of the Water Bend Vibration. *Phys. Rev. Lett.* **2021**, *127*, 116001.

(2) Naskar, B.; Dey, A.; Moulik, S. P. Counter-ion effect on micellization of ionic surfactants: a comprehensive understanding with two representatives, sodium dodecyl sulfate (SDS) and dodecyltrimethylammonium bromide (DTAB). *J. Surfactants Deterg.* **2013**, *16*, 785–794.

(3) Sengupta, S.; Versluis, J.; Bakker, H. J. Observation of a Two-Dimensional Hydrophobic Collapse at the Surface of Water Using Heterodyne-Detected Surface Sum-Frequency Generation. *J. Chem. Phys. Lett.* **2023**, *14*, 9285–9290.

(4) Nishimura, S.; Scales, P. J.; Biggs, S.; Healy, T. W. An electrokinetic study of the adsorption of dodecyl ammonium amine surfactants at the muscovite mica- water interface. *Langmuir* **2000**, *16*, 690–694.

(5) Optics Handling and Care Tutorial. Accessed 2023-10-19. `https://www.thorlabs.com/newgrouppage9.cfm?objectgroup_id=9025`.

(6) Antalicz, B.; Versluis, J.; Bakker, H. J. Observing Aqueous Proton-Uptake Reactions Triggered by Light. *J. Am. Chem. Soc.* **2023**, *145*, 6682–6690.

(7) Venyaminov, S. Y.; Prendergast, F. G. Water (H_2O and D_2O) molar absorptivity in the 1000–4000 cm^{-1} range and quantitative infrared spectroscopy of aqueous solutions. *Anal. Biochem.* **1997**, *248*, 234–245.

(8) Stancik, A. L.; Brauns, E. B. A simple asymmetric lineshape for fitting infrared absorption spectra. *Vib. Spectrosc.* **2008**, *47*, 66–69.

(9) Nihonyanagi, S.; Yamaguchi, S.; Tahara, T. Direct evidence for orientational flip-flop of water molecules at charged interfaces: A heterodyne-detected vibrational sum frequency generation study. *J. Chem. Phys.* **2009**, *130*.

(10) Fu, L.; Chen, S.-L.; Wang, H.-F. Validation of spectra and phase in sub-1 cm^{-1} resolution sum-frequency generation vibrational spectroscopy through internal heterodyne phase-resolved measurement. *J. Chem. Phys. B* **2016**, *120*, 1579–1589.

(11) Kischkat, J.; Peters, S.; Gruska, B.; Semtsiv, M.; Chashnikova, M.; Klinkmüller, M.; Fedosenko, O.; Machulik, S.; Aleksandrova, A.; Monastyrskyi, G. et al. Mid-infrared optical properties of thin films of aluminum oxide, titanium dioxide, silicon dioxide, aluminum nitride, and silicon nitride. *Appl. Opt.* **2012**, *51*, 6789–6798.

(12) Lin, S. H.; Villaeys, A. A. Theoretical description of steady-state sum-frequency generation in molecular adsorbates. *Phys. Rev. A* **1994**, *50*, 5134.

(13) Moll, C. J.; Meister, K.; Versluis, J.; Bakker, H. J. Freezing of Aqueous Carboxylic Acid Solutions on Ice. *J. Chem. Phys. B* **2020**, *124*, 5201–5208.

(14) Moll, C. J.; Versluis, J.; Bakker, H. J. Direct observation of the orientation of urea molecules at charged interfaces. *J. Chem. Phys. Lett.* **2021**, *12*, 10823–10828.

(15) Tang, C. Y.; Allen, H. C. Ionic binding of Na^+ versus K^+ to the carboxylic acid headgroup of palmitic acid monolayers studied by vibrational sum frequency generation spectroscopy. *J. Phys. Chem. A* **2009**, *113*, 7383–7393.

(16) Hirose, C.; Akamatsu, N.; Domen, K. Formulas for the analysis of the surface SFG spectrum and transformation coefficients of cartesian SFG tensor components. *Appl. Spectrosc.* **1992**, *46*, 1051–1072.

(17) Hirose, C.; Akamatsu, N.; Domen, K. Formulas for the analysis of surface sum-frequency

generation spectrum by CH stretching modes of methyl and methylene groups. *J. Chem. Phys.* **1992**, *96*, 997–1004.

(18) Wang, H.-F.; Gan, W.; Lu, R.; Rao, Y.; Wu, B.-H. Quantitative spectral and orientational analysis in surface sum frequency generation vibrational spectroscopy (SFG-VS). *Int. Rev. in Phys. Chem.* **2005**, *24*, 191–256.

(19) Korotkevich, A. A.; Moll, C. J.; Versluis, J.; Bakker, H. J. Molecular Orientation of Carboxylate Anions at the Water–Air Interface Studied with Heterodyne-Detected Vibrational Sum-Frequency Generation. *J. Chem. Phys. B* **2023**, *127*, 4544–4553.

(20) Sun, J.; Bousquet, D.; Forbert, H.; Marx, D. Glycine in aqueous solution: solvation shells, interfacial water, and vibrational spectroscopy from ab initio molecular dynamics. *J. Chem. Phys.* **2010**, *133*.

(21) Malmberg, C.; Maryott, A. Dielectric constant of water from 0 to 100 C. *J. Res. Natl. Bur. Stand.* **1956**, *56*, 1–8.
