# Supplementary material for: Orientational Behavior and Vibrational Response of Glycine at Aqueous Interfaces
Source: J Phys Chem Lett. 2024 Feb 15;15(7):2075–81. doi: 10.1021/acs.jpclett.3c02930 (PMC10895693; doi:10.1021/acs.jpclett.3c02930)
Supplement: Supplementary file 2 — jz3c02930_si_002.pdf [file jz3c02930_si_002.pdf]

jz-2023-02930r.R1

Name: Peer Review Information for "Orientational Behavior and Vibrational Response of Glycine at Aqueous Interfaces"

First Round of Reviewer Comments

Reviewer: 1

Comments to the Author

The authors performed the heterodyne-detected SFG measurement at the water-air interface with the for the small molecule of glycine. The target frequency probes the COO stretch mode which is new and is interesting. The orientation of the glycine molecule was investigated through the intensity of the symmetric and anti-symmetric COO stretch mode with crude approximation (*1). They could not explain the observed SFG intensity of the anti-symmetric mode, and therefore they speculated whether the quadrupole contribution is dominant, but without any convincing data set.

Overall, although I found that probing the COO- with the heterodyne measurement is exciting and the target is relevant to many chemical/biological interfaces, I do not think that the analysis is properly done and the conclusion is very questionable. As such, I cannot recommend the editor to accept the manuscript at the present stage.

1. The first criticism is the assumption the angular distribution of theta is narrow enough to be approximated to the delta function (*1). The authors referred to the paper of SFG analysis (Ref. 45), but I would like to point that the orientation distribution of the free O-H group/hydrophobic CH3 (Phys. Rev. B 59, 12632 (1999),) should be narrower than that of the glycine. When the free O-H is tilted, it will be a H-bonded O-H, no longer free O-H. As such, the angular distribution is narrower. The hydrophobic tail of CH3 is likely repulsive to water and thus it tends to point out to the air. Thus, the distribution could be narrower. In contrast, a glycine molecule has more hydrogen bond acceptor and donors and the distribution should be broader than those of the free O-H or hydrophobic tail. In fact, a combined SFG and simulation work of formic acid at the water-air interface (J. Chem. Phys. 156, 094703 (2022)) shows a very broad orientation, implying that the authors' assumption is likely untrue. If the authors think that the orientational distribution can be approximated to be delta-function, the authors should show some evidences. I suspect that this crude approximation of the delta-function like orientation may deviate the theoretical prediction from the experimental data for the anti-symmetric mode.

2. The second criticism is that the authors connected the origin of the signal with the quadrupole contribution without checking the consistency of their observation for the other vibrational mode (*2)

and/or without checking the other polarization signals such as sps/ppp (*3). For (*2), CH2 can be very easily probed and the authors can somehow check the consistency of their orientation with the CH2 signal. This type of multi-mode probe is not uncommon (see, for example, J. Phys. Chem. B 110, 1727 (2006)). For (*3), the different polarization could provide direct evidence for the quadrupole contribution, like Ref. 54, but no such investigation is done, if my understanding is correct. Without these data, I do not support the authors' view.

3. The conclusion is unnecessarily long, in my opinion.

Reviewer: 2

Comments to the Author

Antalicz studied the zwitterionic state of glycine at the positively and negatively charged surfactant monolayers/water interfaces and air/water interfaces using heterodyne-detected vibrational sum-frequency generation spectroscopy. They carefully examined the Im chi(2) spectra in the fingerprint region as well as the effect of the excess salts on them. Interestingly, glycine shows only a small band due to the asymmetric stretch of COO- at the air/water interface, which they attributed to the signal due to the quadrupolar mechanism, reflecting the random orientation of the glycine at the interface. They subtracted this amplitude from the corresponding bands at the charged surfactant monolayers/water interface to obtain the COO- asymmetric stretch amplitude of the dipole mechanism origin, and evaluated the orientational angle of glycine at each interface through the amplitude ratio between the symmetric and antisymmetric bands of COO-. Based on the results, they concluded that the glycine molecule has substantially different orientational angles, depending on the distance from the charged interface and the difference in the interaction with the surfactant at the interface.

This is a good HD-VSFG study of a solute molecule at aqueous interfaces in the case that the vibrational bands appear with not only the dipole mechanism but also the quadrupole mechanism. The experiments and analysis have been carefully executed, and the authors succeeded in disentangling the observed results although they look very complicated because of the coexistence of two mechanisms of VSFG as well as the contributions of the molecules in different depths. I do not think that the glycine orientation at the interface can attract the interest of physical chemists in various fields but this paper reports prototypical VSFG spectra and analysis in case both the dipole and quadruple mechanisms give rise to the signal. Therefore, I can recommend the publication of this paper in J. Phys. Chem. Lett after the authors make relevant revisions for the issues I list below.

1.

The authors subtract the asymmetric stretch band observed at the air/water interface from other spectra to remove the quadrupolar contribution, considering the quadrupolar contributions in all spectra are the same. However, it is an assumption because the magnitude of the quadrupolar contribution can change with the change of interface, in general. The authors should add some comments on this point.

2.

They evaluated the orientational angle of glycine near the interface by the addition of the excess salt and argued the orientational angle of glycine directly interacting with the surfactant is different from those oriented by the electric field in a deeper region. I agree that this is a plausible interpretation but, if so, we can anticipate relevant vibrational frequency shifts, reflecting the difference in the interaction. Why don't we observe any vibrational frequency change with the addition of the excess salt? The authors need to discuss this point in the paper.

3.

Their discussion on glycine near and far from the charged interface reminds me of the electric double layer (EDL) structure of the charged interface: EDL is considered to consist of the compact Stern layer and diffuse GC layer. I encourage the authors to discuss their conclusion, relating to the EDL structure of the charged interfaces. Is it OK to consider that the difference they observed for glycine is the difference between the structure of the Stern layer and the GC layer?

Minor points:

4. On page 3, right collum, Line 5 from the bottom,

The authors write, "the bulk quadrupolar HD-VSFG response", but this term is misleading because there is the quadrupolar HD-VSFG response originating from the interface region but providing the bulk value. (See ref. 54, for example.) I suggest removing the word "bulk" in this phrase.

5.

In this paper, the authors use expressions such as "Aas : As = -0.43." I suggest changing them to "Aas / As = -0.43."

Author's Response to Peer Review Comments:

Dear Professor Editor,

We hereby would like to submit the revised version of our manuscript *jz-2023-02930r*, entitled "*Orientational Behavior and Vibrational Response of Glycine at Aqueous Interfaces*".

We thank you for your careful handling of our manuscript and the reviewers for their useful suggestions for improvement. In this revised version, we have addressed all their suggestions and implemented the suggested improvements. We have also reformatted the manuscript according to the requested non-scientific changes and journal guidelines. Below you will find our detailed replies to the comments and suggestions made by the reviewers, in which we also explain how we changed the manuscript accordingly.

With kind regards,

Balázs Antalicz, Sanghamitra Sengupta, Aswathi Vilangottunjalil, Jan Versluis and Huib J. Bakker

15th of December 2023, Amsterdam

Itemized response follows on the next pages

Technical note to the editor

In certain discussions, we use indexed references of the manuscript, as well as page numbers (e.g. P1), column designations (left/right column: LC/RC) and line numbers (e.g. L1). We derive these from the original proof version of our manuscript.

To help track changes between the original and the updated versions of our manuscripts, we additionally upload a change-highlighted copy of the new version. In this copy, we additionally mark each change with the corresponding ID, e.g. Reviewer X, Question Y. Changes that are shorter than 1 word (i.e. a few characters) are not highlighted; but are instead described here.

Editor's requests

Request 1

Please submit your publication files without any markups. Any copies that contain highlights, colored text, or tracked changes should be submitted as "Supporting Information for Review Only".

Actions taken

We updated the Latex manuscript file to use journal-appropriate formatting settings, as defined by the Latex style template provided by ACS. We then uploaded this freshly compiled version.

Request 2

TOC Graphic: Please resize the TOC graphic per journal guidelines (2 in x 2 in) and move to the correct position (on the same page as the abstract).

Actions taken

We updated the TOC figure layout to comply with the guidelines.

Request 3

References: In both the main file and the supporting information, fix the style of all references to use JPCL formatting (check all references carefully).

****JPC Letters reference formatting requires that journal references should contain:*

- *around numbers,*
- *author names,*
- *article title (titles entirely in title case or entirely in lower case),*
- *abbreviated journal title (italicized),*
- *year (bolded),*
- *volume (italicized),*
- *and pages (first-last)*

Book references should contain

- *author names,*
- *book title (in the same pattern),*
- *publisher,*
- *city,*
- *and year*

Websites must include

- *date of access*

Actions taken

We manually checked and updated each reference we cite, in both the main text and the SI; and deployed abbreviations for all the journal names. Additionally, we also updated reference formatting via the ACS Latex style template.

Request 4

Graphics: One or more of your figure legends includes a citation. Permission is required if you are using another publisher's or copyright owner's figures/tables verbatim, adapting or modifying them, or using them in part. Permission may not be required if you are only using data to create your figure. If this is the case, please notify our office. Additionally, permission is not required when images are reused from ACS publications.

In all cases of reused or adapted images, even those from papers published open access or in ACS publications, you must cite the source with a credit line in the figure legend. Please use the following format: "Reproduced/adapted from [REFERENCE #]. Copyright [YEAR] [Publisher/Copyright holder]."

You must upload all required permissions documents as individual PDF files with the designation "Other files for Editors only". Please ensure each document is named for the corresponding graphic (e.g. "Permissions-Figure 1"). If the document list is extensive, you may consider compressing the files and uploading a ".zip" file as "Other files for Editors only". If permissions are not accessible, then the graphics need to be redrawn, replaced, or removed. For details, see: https://pubs.acs.org/page/copyright/permissions_otherpub.html.

Response

The caption of Figure 2 (b) reads: '*Relative* $Im\left(\chi^{(2)}\right)$ *contribution (**a**) of the two main carboxylate modes of zwitterionic glycine, based on earlier works. (Ref. 43–46)'*. We cite these earlier works, because we used the reported results to derive the displayed trends in Figure 2 (b). In the main text, we additionally discuss these results and their relevance to our work; see P2/RC/L14-P3/LC/L14. Because we do not reuse or reproduce figures from other works, but merely use the published equations/data, we do not require permissions before publishing.

Actions taken

We updated the figure caption with more concise language: instead of '*based on earlier works*', the caption now says *'derived using the theoretical and experimental results of earlier works'.*

Reviewer 1

Comment 1

*The first criticism is the assumption the angular distribution of theta is narrow enough to be approximated to the delta function (*1). The authors referred to the paper of SFG analysis (Ref. 45), but I would like to point that the orientation distribution of the free O-H group/hydrophobic CH_3 (Phys. Rev. B 59, 12632 (1999),) should be narrower than that of the glycine. When the free O-H is tilted, it will be a H-bonded O-H, no longer free O-H. As such, the angular distribution is narrower. The hydrophobic tail of CH_3 is likely repulsive to water and thus it tends to point out to the air. Thus, the distribution could be narrower. In contrast, a glycine molecule has more hydrogen bond acceptor and donors and the distribution should be broader than those of the free O-H or hydrophobic tail. In fact, a combined SFG and simulation work of formic acid at the water-air interface (J. Chem. Phys. 156, 094703 (2022)) shows a very broad orientation, implying that the authors' assumption is likely untrue. If the authors think that the orientational distribution can be approximated to be delta-function, the authors should show some evidences. I suspect that this crude approximation of the delta-function like orientation may deviate the theoretical prediction from the experimental data for the anti-symmetric mode.*

Response

We thank the Reviewer for raising this point on the width of the angular distribution in different environments. We would like to point out that we only used the delta angular distribution for calculating the orientation of glycine at charged surfaces; not at the neat water/air interface.

To understand the zwitterion's behavior at the neat water/air interface, we first consider its solvation properties. As pointed out by the Reviewer, the zwitterion has multiple hydrogen-bond acceptor and donor sites. This means that ~7-8 water-molecules are required stabilize its solvation shell, per the MD results of Ref. 15. As a result of this well-solvated character, zwitterions are likely not present at the neat water/air surface in a large concentration. Because of its solvation properties, we fully agree with the Reviewer, that zwitterions at the neat water/air interface should have a very broad angular distribution. To apply the theoretical predictions from Figure 2 to the neat water-air interface, we would need to integrate over a very broad angular range; resulting in overall very small dipolar HD-VSFG contributions. In the manuscript, we thus concluded that "*This also means that zwitterions have no dipolar HD-VSFG contributions at the neat water/air interface, meaning that they do not have a net orientation in absence of surfactants and electric fields.*" (P3/RC/L56 – P4/LC/L1). This is in agreement with the conclusions of the Reviewer.

Next, we consider the case of charged interface. Here, the orienting effects do not result from the solvation energy penalty of a hydrophobic moiety, but arise due to direct interactions with the surfactants and strong electric fields near the surface. Because of the geometrical constraints of direct interactions, and because of the strength of the electric field near the surface, a charged interface will lead to much more narrow angular distributions than at the air/water interface.

Inspired by the comment of the reviewer, we decided to use a less crude approximation for the angular distribution for the case of the field-oriented glycine species. For this, we now use a thermodynamics-based model, as explained in a new section of the section of the SI. Using this model, we get $a_{as}/a_s = -0.24$ for any ionic strength. This ratio is overall very similar the $a_{as}/a_s = -0.2$ ratio predicted by the delta-distribution assumption.

Actions taken

- We updated the paragraph on P3/LC/L7-12, to include: "... *In general, the narrow distribution assumption is sufficiently accurate for glycine species oriented by direct surfactant-interactions or by strong electric fields. In the SI, we show that in case of field-oriented zwitterions, the thermodynamics-based calculations yield similar* $\boldsymbol{a_s}/\boldsymbol{a_{as}}$ *ratios, see Table 2 (a) and (b).* ...."
- We included a new section in the SI, detailing our derivation and results for the thermodynamics-based model.
- To help the reader throughout the discussion, we use a new notation: $R \coloneqq \boldsymbol{a_s}/\boldsymbol{a_{as}}$.
- We updated Table 2 with the newly obtained R ratios.
- In Table 2, we introduced (a)-(d) naming for the sub-tables.
- We updated the caption of Table 2.
- We also updated the text after Table 2 (discussion and conclusions, P4/LC/L37-P5/LC/L13) wherever $R^{E\downarrow}$ and $R^{E\uparrow}$ ratios are mentioned.
- We include both kind of $R^{E\downarrow}$ and $R^{E\uparrow}$ predictions in the conclusion.
- We updated the sentences on P4/LC/L33-37, to say: *"... We then obtain the average orientation angle θ of the zwitterionic form of glycine near the water surface, by comparing the experimentally obtained ratios with the theoretical values, using the approximation of $\Delta\theta = 0$. For zwitterions oriented by the electric field, we use the R ratios predicted by our thermodynamics-based calculations."*
- Because the updated $R^{E\downarrow} = -0.24$ value is farther away from $R^{DA^+} = -0.18$, we updated the discussion on P4/LC/L45-L55 to include possible COO^--interacting zwitterions: "*The observed differences can be explained if we consider the emergence of zwitterions with a COO^- group that coordinate with the monolayer ($R^{COO^-} = 0$, see Figure 2 (f)), which would then shift the R^{DTA^+} ratio to more positive values.*"
- With $R^{DA^+} = -0.28$, the difference to $R^{E\downarrow} = -0.24$ has decreased and is now within the experimental error of these ratios. This means that a much smaller contribution is required from zwitterions with singly coordinating COO^- groups. As such, we moved their illustration from the TOC to SI Figure 17.
- In connection to this, we improved the wording and shortened the paragraph on P4/RC/L26-L37. This paragraph details why adding NaCl could decrease the HD-VSFG contributions of zwitterions with singly coordinating COO^- groups. To help ease the flow of the discussion, we also merged the paragraph with the preceding one.

Comment 2

*The second criticism is that the authors connected the origin of the signal with the quadrupole contribution without checking the consistency of their observation for the other vibrational mode (*2) and/or without checking the other polarization signals such as sps/ppp (*3). For (*2), CH_2 can be very easily probed and the authors can somehow check the consistency of their orientation with the CH_2 signal. This type of multi-mode probe is not uncommon (see, for example, J. Phys. Chem. B 110, 1727 (2006)). For (*3), the different polarization could provide direct evidence for the quadrupole contribution, like Ref. 54, but no such investigation is done, if my understanding is correct. Without these data, I do not support the authors' view.*

Response

The Reviewer would like us to show further proof of our assignment to the quadrupolar contribution of the $\nu_{as}^{COO^-}$ vibration.

To address this comment, we first repeat our assignment in the manuscript. In the case of a $D(T)A^+$-covered surfaces with 1 M NaCl added, we observe negative $\boldsymbol{a_s}$ and $\boldsymbol{a_{as}}$ values; and for the neat water/air interface, we observe zero $\boldsymbol{a_s}$ and negative $\boldsymbol{a_{as}}$ values. We conclude, that *"This behavior cannot be easily explained using the theoretical framework presented before*" (P3/RC/L40-41). As a result, we note that "*We therefore consider that the observed $\boldsymbol{a_{as}}$ values are very similar for the neat water/air interface and for 1 M NaCl solutions covered with D(T)A+ monolayers. Due to its apparent insensitivity to electric fields, we infer that this small negative $\boldsymbol{a_{as}}$ does not have a dipolar origin."* (P3/RC/L41-46).

The Reviewer also suggests other methods to support our assignment of the quadrupolar contribution by the $\nu_{as}^{COO^-}$ mode of the zwitterion. One of these methods is to measure HD-VSFG signals in other polarization configurations (e.g. SPS, PPP), like in Ref. 54. In this article, the authors demonstrate that different quadrupolar mechanisms come into effect in different polarizations. This distinction between different types of quadrupolar contributions was enabled by the unique symmetries (D_{6h}) of the studied benzene molecule, which resulted in negligible/forbidden dipolar contributions. Although Ref. 54 is an extremely nice and interesting article, it does not present a method or approach to distinguish dipolar and quadrupolar contributions for systems that a priori can have both, e.g. the strongly dipolar glycine zwitterion.

An alternative method suggested by the Reviewer is to perform multi-mode probing; in particular by studying the vibrations of glycine's CH_2 moiety. We already account include this: in addition to the studied $\nu_{s/as}^{COO^-}$ modes, we observe the zwitterion's in-plane ω^{CH_2} vibration at 1324 cm^{-1}; see Table 1. In the manuscript, we write that the HD-VSFG signal of this vibration closely follows the behavior of the $\nu_s^{COO^-}$ vibration: *"Because their orientation flips, so does the sign of the SFG contributions of both the $\nu_s^{COO^-}$ and the in-plane ω^{CH_2} vibrations"* (P3/LC/L19) and that *"Overall, the behavior of the $\nu_s^{COO^-}$ and ω^{CH_2} bands is consistent with the observations in Figure 3, where the increased ionic screening led to a decrease of the near-surface electric field and thus the net glycine HD-VSFG signal."* (P3/RC/L31). In Figure 1, 3 and SI Figure 12, we show that the ω^{CH_2} signal follows $\nu_s^{COO^-}$ signal even at the neat water/air interface. We thus assess that ω^{CH_2} signals show great consistency with the dipolar HD-VSFG contributions predicted by our interpretation framework.

Comment 3

The conclusion is unnecessarily long, in my opinion.

Response and actions taken

We thank the Reviewer for this suggestion. Following the Reviewers' point, we significantly shortened the concluding paragraph (P5/LC/L17-L25), to say:

> '… *We anticipate that such information can provide a better understanding of glycine's behavior near neural synapses and near glycine-specific receptors, where electric fields and specific interactions both play an important role.*

Reviewer 2

Comment 1

The authors subtract the asymmetric stretch band observed at the air/water interface from other spectra to remove the quadrupolar contribution, considering the quadrupolar contributions in all spectra are the same. However, it is an assumption because the magnitude of the quadrupolar contribution can change with the change of interface, in general. The authors should add some comments on this point.

Response

We thank the Reviewer for suggesting us to discuss the amplitude of the quadrupolar for different types of interfaces. Using the notation of Ref. 57, quadrupolar contributions originate from the bulk (χ^B: negligible for our experimental setup, see Ref 51) and the surface. The surface-specific contribution has components carrying surface-specific (χ^{IQ}) and bulk-specific information (χ^{IQB}). If the observed quadrupolar response would be highly sensitive to the interfacial properties, we would observe large changes in HD-VSFG signals in Figure 4 and SI Figure 12. In these figures, the zwitterions' dipolar signals are the smallest, while the surface-coverage conditions are exchanged.

- In Figure 4, we show that *"$\boldsymbol{a_{as}}$ values are very similar for the neat water/air interface and for 1 M NaCl solutions covered with D(T)A$^+$ monolayers*." (P3/RC/L42-43).
- In SI Figure 12, we show "*that the small negative $\boldsymbol{a_{as}}$ is not changed when adding salts*" (P3/RC/L53-54).

Because the observed $\boldsymbol{a_{as}}$ results are identical within measurement error, we conclude that the surface-specific χ^{IQ} contributions are negligible. As a result, the quadrupolar response is dominated by χ^{IQB}, making our subtraction method valid.

Actions taken

- We update the sentence on P3/RC/L44-45, to say: *"Due to its apparent insensitivity to electric fields <u>and interfacial properties</u>, ..."*
- We also updated the sentence on P3/RC/L51-52, to say: *"The observed quadrupolar (Ref. 56,57) HD-VSFG contributions often <u>carry bulk information: they are generally insensitive to interfacial properties and scale with solute concentration</u>."*

Comment 2

They [the authors] *evaluated the orientational angle of glycine near the interface by the addition of the excess salt and argued the orientational angle of glycine directly interacting with the surfactant is different from those oriented by the electric field in a deeper region. I agree that this is a plausible interpretation but, if so, we can anticipate relevant vibrational frequency shifts, reflecting the difference in the interactions. Why don't we observe any vibrational frequency change with the addition of the excess salt? The authors need to discuss this point in the paper.*

Response

In Figure 3, we added an increasing amount of NaCl and observed the HD-VSFG response of glycine. In this frequency window, we probe the narrow ω^{CH_2} and $\nu_s^{COO^-}$ peaks, alongside with the broader $\nu_{as}^{COO^-}$ peak that also overlaps with the dipolar δ^{H_2O} mode. Inspired by the comment of the Reviewer, we further analyzed the frequencies of the ω^{CH_2} and the $\nu_s^{COO^-}$ peaks. By zooming in on the figure, we observe that the center of the ω^{CH_2} peak does remain at a constant frequency, while the $\nu_s^{COO^-}$ peak appears to blue-shift by approximately 10 cm^{-1}. Therefore we indeed observe that the addition of the salt not only changes the orientation of the zwitterions, but also the frequency of the $\nu_s^{COO^-}$ vibration. This is most likely a result of a change of the direct environment of this group (e.g. the hydrogen bonding), induced by the addition of salt.

In Figure 4, we also observe some frequency shifts of the $\nu_s^{COO^-}$ signal, when comparing DS-coverage (COO^- points to bulk) and DA^+/DTA^+ coverage (COO^- points to surfactant). $Im(\chi^{(2)})$ features with such a small amplitude, however, are difficult to accurately analyze. This is because the dispersive shape of larger $Re(\chi^{(2)})$ spectra (typical amplitude ~0.3…1 units) can mix even in case of small phasing errors, shifting the apparent peak frequencies and amplitudes. This phase shift can then occur for both the recorded glycine signals and the subtracted surfactant signals (SI Figure 5). Given the relative amplitude uncertainties in Table 2 (d), we consider that in in Figure 4, an accurate analysis of $\nu_s^{COO^-}$ frequencies is limited by our experimental means.

Actions taken

In agreement with the intent of the Reviewer's suggestion, we make note of the observed frequency shift of the $\nu_s^{COO^-}$ vibration in Figure 3. On P3/LC/L55, we now write that:

> '*With the increase of the salt concentration, we additionally observe a small blue-shift of the $\nu_s^{COO^-}$ signal. This blue-shift can be explained if we consider that at higher ionic strengths, the added ions influence the probed zwitterions' solvation environment, and therefore the vibrational frequency of the COO^- group.*

Comment 3

Their discussion on glycine near and far from the charged interface reminds me of the electric double layer (EDL) structure of the charged interface: EDL is considered to consist of the compact Stern layer and diffuse GC layer. I encourage the authors to discuss their conclusion, relating to the EDL structure of the charged interfaces. Is it OK to consider that the difference they observed for glycine is the difference between the structure of the Stern layer and the GC layer?

Response

We thank the reviewer for raising this very interesting point. With charged interfaces at low ionic strengths, we could identify strong HD-VSFG contributions form field-oriented zwitterions; similar to how water shows an orientational response in the diffuse Gouy-Chapman layer. Similarly, at high ionic strengths, we observe a response increasingly dominated by surfactant-specific interactions; which could show parallels to the orientational behavior of water in the Stern-layer. We include this notion in our conclusions section, see below.

Actions taken

- At P5/LC/L4, we write: *"Overall, the field-induced orientational behavior zwitterionic glycine shows similarities to the field-induced orientation of water molecules in the diffuse Gouy-Chapman layer."*
- At P5/LC/L13, we write: *"To continue our previous analogy: at high ionic strengths, this tendency of zwitterions towards specific interactions could show parallels to the behavior of water molecules in the Stern-layer."*

Comment 4 *(minor point)*

On page 3, right column, Line 5 from the bottom, The authors write, "the bulk quadrupolar HD-VSFG response", but this term is misleading because there is the quadrupolar HD-VSFG response originating from the interface region but providing the bulk value. (See ref. 54, for example.) I suggest removing the word "bulk" in this phrase.

Response

We thank the reviewer for pointing out a possible source of confusion regarding semantics. In our work, we aim to use semantics in the most correct way possible. We agree with the Reviewer, that in case of a surface-specific study, the usage of the word 'bulk' must be done with great care.

We believe that the current terminology of quadrupolar contributions could be clearer on the matter. This is best seen if we compare Ref. 54, which discusses a *'quad3 contribution'* that has bulk properties and interface localization; while Ref. 57 discusses *'bulk quadrupole contribution'*, which consists of χ^B (negligible for our experimental setup, see Ref 51); and χ^{IQB}, a '*bulk contribution*' that '*reflects no interfacial properties*'.

Regardless of the semantic differences above, both sources agree on that the quadrupolar contribution with bulk origin carries bulk information, and that it *'scales with solute concentration'* (P3/RC/L51-52).

Actions taken

In agreement with the Reviewer, we updated the sentence on P3/RC/L54-55 to omit the word 'bulk'.

Comment 5 *(minor point)*

In this paper, the authors use expressions such as "A_{as} : A_s = -0.43." I suggest changing them to "A_{as} / A_s = -0.43."

Response and actions taken

We thank the Reviewer for pointing out a place of possible improvement in our presentation. We now use the suggested way to denote "A_{as} / A_s" and β_{aac} / β_{ccc} ratios.

jz-2023-02930r.R2

Name: Peer Review Information for "Orientational Behavior and Vibrational Response of Glycine at Aqueous Interfaces"

Second Round of Reviewer Comments

Reviewer: 1

Comments to the Author

The authors' reply for my comment 1 is very convincing.

Probably my comment 2 for the quadrupole contribution were not clear to the authors. I meant that, to assign the signal to the quadrupole contribution, researchers have been more careful by using 1. Polarization-dependent HD-SFG (Ref. 54), 2. Computation (Ref. 53), 3. Systematic study of relevant molecules (Ref. 55). In contrast, the authors seem to attribute the signal to the quadrupole contribution rather straightforwardly without doing additional analysis. Given that the quadrupole contribution is highly under debate (see *) and therefore other research groups use much more careful assessment for the quadrupole contribution, I would be more careful to assign the signal to quadrupole. Probably, considering the following points would be useful for thinking about the quadrupole contribution.

1. Why is the quadrupole contribution comparable with the dipole contribution for COO- vibration mode? COO- can have a large transition dipole moment and I would be surprised that COO- transition quadrupole contribution is even larger than the COO- transition dipole contribution. Benzene (or benzene derivatives) are very rare case of the large (transition) quadrupole, but generally the dipole contribution is much stronger than the quadrupole contribution. Probably, some calculation would be helpful (Just simple ab initio calculation), if the authors really believe that the transition quadrupole moment is so large for COO- and the transition dipole is so small.

2. The authors indicate that the positive/negative sign of the symmetric/antisymmetric mode predicted by the model (Fig. 2b) suggests the quadrupole contribution. If my understanding is correct, the plane of the molecules was set to be parallel to the surface normal. When this is not parallel (which is more probable), is this negative/positive sign relation for the symmetric/anti-symmetric still valid? Probably, the authors should make the two-dimensional plot, instead of one-dimensional plot of SFG sign vs. theta (Fig. 2b). I think that the situation of one mode being zero and the other mode being

negative is possible, if the molecular plane is tilted with respect to the surface normal. If so, the data does not suggest the quadrupole contribution.

3. If the quadrupole contribution is not unambiguously identified, I would suggest the authors to tone down their statement.

* Although I suspect that the authors know the debate on the quadrupole contribution, I would like to note it to be sure that we are on the same page. The benzene quadrupole contribution was proposed by Tahara (and benzene derivative by Bonn), but was denied by Shen and Morita. Water bending mode is proposed to be governed by the quadrupole contribution in Tahara, Morita, and your group, while Bonn and Benderskii disagree. Among Tahara and Morita, Tahara and Morita's interpretations are totally orthogonal (interfacial quadrupole vs. bulk quadrupole). The C=O quadrupole contribution was proposed by Morita, but no group supports his statement.

Reviewer: 2

Comments to the Author

This paper after the revision is publishable as it is, in principle. However, the first equation in the second paragraph in page 6 should be, pH = 1/2 (pKa(cation)+pKa(zwitterion))~6.2. It need be corrected.

Author's Response to Peer Review Comments:

Dear Professor Editor,

We hereby would like to submit the revised version of our manuscript *jz-2023-02930r*, entitled "*Orientational Behavior and Vibrational Response of Glycine at Aqueous Interfaces*".

We thank you for your careful handling of our manuscript and Reviewer 1 and 2 for their new suggestions for improvement. We have addressed all suggestions and implemented the suggested improvements. We have also reformatted the manuscript according to the requested non-scientific changes and journal guidelines. Below you will find our detailed replies to the comments and suggestions made by the reviewers, in which we also explain how we changed the manuscript accordingly.

With kind regards,

Balázs Antalicz, Sanghamitra Sengupta, Aswathi Vilangottunjalil, Jan Versluis and Huib J. Bakker

26th of January 2024, Amsterdam

Itemized response follows on the next pages

Editor's requests

Request 1

Author Affiliations: Please include postal codes/country in the author affiliations in the publication file(s).

Actions taken

We included the requested changes.

Request 2

Supporting Information: Please number SI pages in the following format: "S1, S2…"

Actions taken

We incorporated the requested changes into the SI. To conform to this display style, we additionally updated the Figure and Table naming to say 'Figure S9' instead of 'SI Figure 9' and 'Table S1' instead of 'SI Table 1'.

Reviewer 1

Overview comment

The authors' reply for my comment 1 is very convincing.

*Probably my comment 2 for the quadrupole contribution were not clear to the authors. I meant that, to assign the signal to the quadrupole contribution, researchers have been more careful by using 1. Polarization-dependent HD-SFG (Ref. 54), 2. Computation (Ref. 53), 3. Systematic study of relevant molecules (Ref. 55). In contrast, the authors seem to attribute the signal to the quadrupole contribution rather straightforwardly without doing additional analysis. Given that the quadrupole contribution is highly under debate (see *) and therefore other research groups use much more careful assessment for the quadrupole contribution, I would be more careful to assign the signal to quadrupole. Probably, considering the following points would be useful for thinking about the quadrupole contribution.*

Response

We thank the Reviewer for their kind words. In the following we will address the points that are raised by the Reviewer in connection to our assignment of part of the signal to a quadrupolar response.

Comment 1

Why is the quadrupole contribution comparable with the dipole contribution for COO- vibration mode? COO- can have a large transition dipole moment and I would be surprised that COO- transition quadrupole contribution is even larger than the COO- transition dipole contribution. Benzene (or benzene derivatives) are very rare case of the large (transition) quadrupole, but generally the dipole contribution is much stronger than the quadrupole contribution. Probably, some calculation would be helpful (Just simple ab initio calculation), if the authors really believe that the transition quadrupole moment is so large for COO- and the transition dipole is so small.

Response

In steady-state absorption spectroscopy, transition dipoles interact with the electric field while transition quadrupoles interact with gradients in the exciting electric fields. Because quadrupolar transition moments are 10^5…10^8 times smaller, quadrupolar transitions are extremely hard to detect in conventional absorption spectroscopy; see [A. Campargue et al, Observation of electric-quadrupole infrared transitions in water vapor, *Phys. Rev. Lett*. **2020**].

In HD-VSFG spectroscopy, the dipolar response originates from well-oriented transition dipoles at or near the surface, such as directly interacting and field-oriented zwitterions. At the same time, interfacial quadrupolar contributions – (see Ref. 57 and the previous Comment 1 of Reviewer 2) – are non-negligible, because the exciting electric fields show strong gradients near the surface. This leads to a quadrupolar response that can have an amplitude similar to the dipolar response. In general, quadrupolar contributions do not require orienting effects, and scale with the concentration of the molecules carrying the probed vibrations and the field gradients. Recent experimental works demonstrated that the magnitude of dipolar and quadrupolar contributions can indeed be similar, see Refs. 47, 51, and 52.

Comment 2

The authors indicate that the positive/negative sign of the symmetric/antisymmetric mode predicted by the model (Fig. 2b) suggests the quadrupole contribution. If my understanding is correct, the plane of the molecules was set to be parallel to the surface normal. When this is not parallel (which is more probable), is this negative/positive sign relation for the symmetric/anti-symmetric still valid? Probably, the authors should make the two-dimensional plot, instead of one-dimensional plot of SFG sign vs. theta (Fig. 2b). I think that the situation of one mode being zero and the other mode being negative is possible, if the molecular plane is tilted with respect to the surface normal. If so, the data does not suggest the quadrupole contribution.

Response

We agree with the Reviewer that the zwitterion's plane is not fixed to the surface normal. In our description we implicitly include that the C-C-N molecular plane can be rotated away from the surface normal (tilt angle). We also account for the angle of the COO^- group's rotation around the C-C bond (P8/L25). Following Refs 45 and 46, we use expressions for the signal amplitudes that only depend on $\theta\theta$, the '*angle between the surface normal and the COO^- group's symmetry axis*' (P7/L38-39). Our approach is detailed additionally in Supplementary Information.

We recognize that Figure 2 (a) might have been confusing in this respect, so we updated this illustration by including a strongly tilted molecular C-C-N plane. We would like to point out that the special cases shown in Figure 2 (c) – (e) do have a surface-perpendicular C-C-N plane because this corresponds to the definition of these special cases.

Based on the above conclusions and our results in the main text and the SI, we find that physically relevant angular distributions of glycine zwitterions cannot yield dipolar contributions where $aa_{ss}=0$ and $aa_{aass}<0$.

Actions taken

To clarify the modeling, we updated Figure 2 (a) to show a zwitterion with a heavily tilted molecular C-C-N plane, and emphasize that the COO^- group can freely rotate around its symmetry axis.

Comment 3

If the quadrupole contribution is not unambiguously identified, I would suggest the authors to tone down their statement.

Response

Our observations, together with the results of previous related experimental and theoretical studies, constitute strong evidence for the quadrupolar nature of part of the observed signals. Nevertheless, following the advice of the reviewer, we toned down the wording about the assignment.

Actions taken

On Page 11, Lines 7-10 we write "… *we **tentatively** assign this contribution to the quadrupolar HD-VSFG response of the anti-symmetric stretching vibration… ".*

On Page 14, Line 15, we write: "… *we find **strong evidence** that the antisymmetric stretching vibration has a quadrupolar HD-VSFG contribution, which is independent of electric fields…*" **Reviewer 2**

Comment 1

This paper after the revision is publishable as it is, in principle. However, the first equation in the second paragraph in page 6 should be, pH = 1/2 (pKa(cation)+pKa(zwitterion))~6.2. It need be corrected.

Response and actions taken

We thank the Reviewer for pointing out this text error. We implemented the suggested change.
